# Distribution patterns of phthalic acid esters in soil particle-size fractions determine biouptake in soil-cereal crop systems

**DOI:** 10.1038/srep31987

**Published:** 2016-08-24

**Authors:** Wenbing Tan, Yuan Zhang, Xiaosong He, Beidou Xi, Rutai Gao, Xuhui Mao, Caihong Huang, Hui Zhang, Dan Li, Qiong Liang, Dongyu Cui, Akram N. Alshawabkeh

**Affiliations:** 1 State Key Laboratory of Environmental Criteria and Risk Assessment, Chinese Research Academy of Environmental Sciences, Beijing 100012, China; 2 State Environmental Protection Key Laboratory of Simulation and Control of Groundwater Pollution, Chinese Research Academy of Environmental Sciences, Beijing 100012, China; 3 Hebei Provincial Academy of Environmental Sciences, Shijiazhuang 050037, China; 4 School of Resource and Environmental Science, Wuhan University, Wuhan 430079, China; 5 Lanzhou Jiaotong University, Lanzhou 730070, China; 6 College of Plant Science and Technology, Beijing University of Agriculture, Beijing 102206, China; 7 Civil and Environmental Engineering Department, Northeastern University, Boston Massachusetts 02115, United States

## Abstract

The use of wastewater irrigation for food crops can lead to presence of bioavailable phthalic acid esters (PAEs) in soils, which increase the potential for human exposure and adverse carcinogenic and non-cancer health effects. This study presents the first investigation of the occurrence and distribution of PAEs in a maize-wheat double-cropping system in a wastewater-irrigated area in the North China Plain. PAE levels in maize and wheat were found to be mainly attributed to PAE stores in soil coarse (250–2000 μm) and fine sand (53–250 μm) fractions. Soil particle-size fractions with higher bioavailability (i.e., coarse and fine sands) showed greater influence on PAE congener bioconcentration factors compared to PAE molecular structures for both maize and wheat tissues. More PAEs were allocated to maize and wheat grains with increased soil PAE storages from wastewater irrigation. Additional findings showed that levels of both non-cancer and carcinogenic risk for PAE congeners in wheat were higher than those in maize, suggesting that wheat food security should be prioritized. In conclusion, increased soil PAE concentrations specifically in maize and wheat grains indicate that wastewater irrigation can pose a contamination threat to food resources.

Phthalic acid esters (PAEs or phthalates) are commonly used plasticizers found to act as endocrine disrupting compounds^1^ that may induce carcinogenic, teratogenic, and mutagenic effects^2^,^3^,^4^. Specific PAEs, such as bis(2-ethylhexyl) phthalate (DEHP), butyl benzyl phthalate (BBP), di-n-butyl phthalate (DnBP), di-n-octyl phthalate (DnOP), diethyl phthalate (DEP), and dimethyl phthalate (DMP) are considered priority pollutants by the United States Environmental Protection Agency (USEPA)^5^. Approximately 6.0 million tons of PAEs are produced and consumed annually worldwide according to the estimation by Arbeitsgemeinschaft PVC and UMWELT e.V (AgPU). Given the large production and widespread application of PAEs-containing products, PAE residues have been routinely detected in various environmental matrices such as soils^6^,^7^, plants^8^,^9^, water^10^, sediments^11^, and air^12^.

There is a growing concern that edible crops may absorb residual PAEs in agricultural soils. PAEs are subsequently transferred into the food supply chain^2^,^13^,^14^, increasing the potential for adverse human health impacts. Human exposure to PAEs is considered widespread due to their extensive use. Dietary intake from contaminated food is the primary source of PAE exposure in the general population^15^; according to the model of the European Union System for the Evaluation of Substances (EUSES), the exposure to edible crops can account for 89.9% and 51.0% of the total human intake exposure for dibutyl phthalate (DBP) and DEHP, respectively^16^. Previous studies reported the occurrence and status of PAEs in the soil-crop system and showed that the accumulations of PAEs in plant tissues are mainly govern by combined factors of organic matter, minerals, pH, temperature and microorganisms of soils and intrinsic physicochemical properties of pollutants^8^,^17^,^18^,^19^,^20^,^21^. However, most of these studies were conducted in vegetable crop fields, whereas the dynamics of PAEs in soil–cereal crop systems, particularly the cereal cropping system from actual agricultural fields, remains unclear. Globally, maize and wheat serve as the first and third most important cereal crops, respectively, accounting for approximately 54% of cultivated areas and 56% of yields for cereal crops in China^22^. Given the scarcity of fresh water resources, an increasing area of agricultural land growing wheat and maize is irrigated with wastewater in China, particularly in the North China Plain^23^, and the area of wastewater irrigation in China is over 360,0000 ha according to the second national wastewater irrigation survey. With limited fresh water, wastewater may serve as an ideal option for agricultural irrigation because of its nutritive value and potential to enhance soil quality^24^. However, wastewater is also a source of chemicals of emerging concerns (CECs) including PAEs, ultimately resulting in contamination of agricultural soils and crops^25^,^26^. Clearly, investigating the dynamics of PAEs in a soil–maize/wheat system irrigated with wastewater is of significance due to the fact that freshwater resources continue to decrease and turning to use of treated and untreated wastewater for irrigation in agricultural systems gradually gains prevalence.

The form and behavior of hydrophobic organic pollutants in soils are determined by their chemistry and chemical interactions with soil minerals and organic matter. Soil is a microcosm in which various factors, such as soil organic matter (SOM), clay minerals and biogeochemical process, may significantly impact the dynamics of hydrophobic organic pollutants in soils^27^. Soil particle-size fractionation can be used to distinguish pools of different soil compositions and the targeting pollutants^28^,^29^, allowing the investigation on the processes controlling the occurrence, bioavailability and transfer of pollutants in a soil-plant system. It is evidenced that organic matter bound to silt and clay fractions is more stable than that associated with the sand fraction^30^. Given the hydrophobic organic pollutants are mainly sorbed to organic matter in soils, the hydrophobic organic pollutants in sand fraction may be more readily released to soil solution with the degradation of organic matter than those in other soil fractions and may thus potentially exert more chance to be taken up by plants^31^. To our knowledge, there is little information on assessing the biouptake of PAEs in a maize-wheat double-cropping system irrigated by wastewater based on the partition of PAEs in different soil particle-size fractions. In this study, the correlation between distribution patterns of PAEs in soil and accumulation of PAEs in maize and wheat were investigated, and the bioavailability and environmental risks of PAEs were accordingly evaluated.

## Results and Discussion

### Occurrences of PAEs in bulk soil and soil particle-size fractions

No significant differences in concentrations of the 14 target PAE congeners were found between the two batches of soil samples collected in October 2012 and June 2013 (Supplementary Fig. S1), indicating a balance of soil PAE congener concentration between the rate of PAE input and the rate of PAE output at any one time. Accordingly, average values of the soil PAE data collected at different times were used. PAE concentrations and detection frequencies in the soil samples are shown in Supplementary Tables S2 and S3, respectively. The PAE concentrations in the soil from the area irrigated by wastewater were significantly higher than those in the reference adjacent agricultural soil irrigated by groundwater (Supplementary Table S4). In addition, the PAE levels in the wastewater (Supplementary Table S5) were significantly higher than those in the groundwater (Supplementary Table S5) and the reported PAE levels in river waters^32^,^33^.

**Figure 1:**
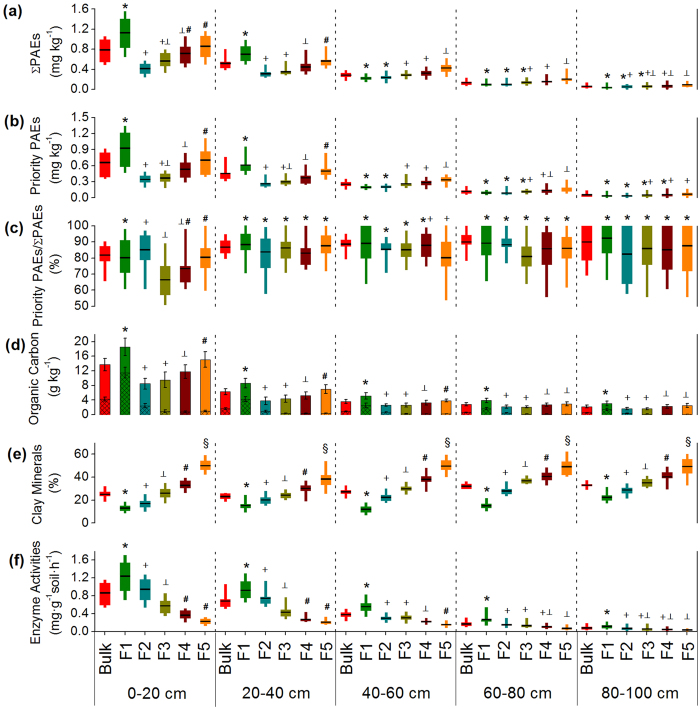
Concentrations of ΣPAE (**a**), total concentrations of the six priority PAEs (**b**), percentages of six priority PAEs in ΣPAE (**c**), organic carbon contents (**d**), percentages of clay minerals (**e**), and enzyme activities (**f**) in bulk soils and soil particle-size fractions in different soil layers. In (**a**)–(**c**) and (**e**,**f**), thin vertical line represents 10th and 90th percentiles. Box represents 25th and 75th percentiles, and central horizontal line represents mean. In (**d**), the column filled with oblique grids represents active organic carbon. In the x axis, bulk means bulk soil, F1, F2, F3, F4, and F5 represent soil coarse sand (250–2000 μm), fine sand (53–250 μm), coarse silt (20–53 μm), fine silt (2–20 μm), and clay (<2 μm), respectively. Means followed by the same footnote symbol(s) for each soil layer are not significantly different at *p* < 0.05.

Among the 14 PAEs, DMP, DEP, DnBP, BBP, DEHP, and DnOP considered by the U.S. E.P.A. as the six priority pollutants, were found in soil layers (0–20 cm) at detection frequencies of 96%, 100%, 100%, 12%, 62%, and 100%, respectively. Detection frequencies in soil of all 14 PAEs significantly dropped with depth. DEHP, DnOP, and DnBP showed higher concentrations in all soil layers than the other 11 PAEs, which correspond to their higher concentrations in the irrigation wastewater (Supplementary Table S5). The result was in agreement with other findings that DEHP and DnBP are the dominant PAEs in soils, air, water, and sediment^6^,^34^,^35^,^36^,^37^,^38^. This observation can also be attributed to the fact that DEHP and DnBP are the most commonly produced PAEs^1^,^18^,^39^. All six priority PAE congeners except DEHP had higher average concentrations in the wastewater irrigation area of Wangyanggou than the mean levels in national agricultural soil of China^36^. According to the soil environmental quality standard set by the U.S. E.P.A., DMP exceeded the limit in all soil layers at 0–20 and 20–40 cm depths, in 85% of the soil layers at 40–60 cm depth, and in 54% of the soil layers at 60–80 cm depth. DEP exceeded the limit in all soil layers at 0–20 and at 20–40 cm depths, in 69% of the soil layers at 40–60 cm depth, and in 23% of the soil layers at 60–80 cm depth. DnBP exceeded the limit in all soil layers at 0–20 and 20–40 cm depths, and in 39% of the soil layers at 40–60 cm depth. With increased soil depth, the concentrations of ΣPAE, six priority PAEs, and individual PAE congeners gradually decreased due to the sorption and migration of PAEs with continuous irrigation. It should be noted that the ratio of the six priority PAEs to ΣPAE exhibited an increasing trend from 81.7 ± 7.7% to 90.1 ± 12.1% with soil depth (Fig. 1c), indicating that the six priority PAEs were more mobile relative to the non-priority PAEs, which may be explained by the relatively higher water solubility of DMP, DEP and DnBP. DEHP, with a low water solubility (2.07 μg L^−1^), was hardly detected in deeper soils (see Supplementary Table S2) and thus did not contribute to the higher ratio of six priority PAEs to ΣPAE in deeper soils.

**Figure 2:**
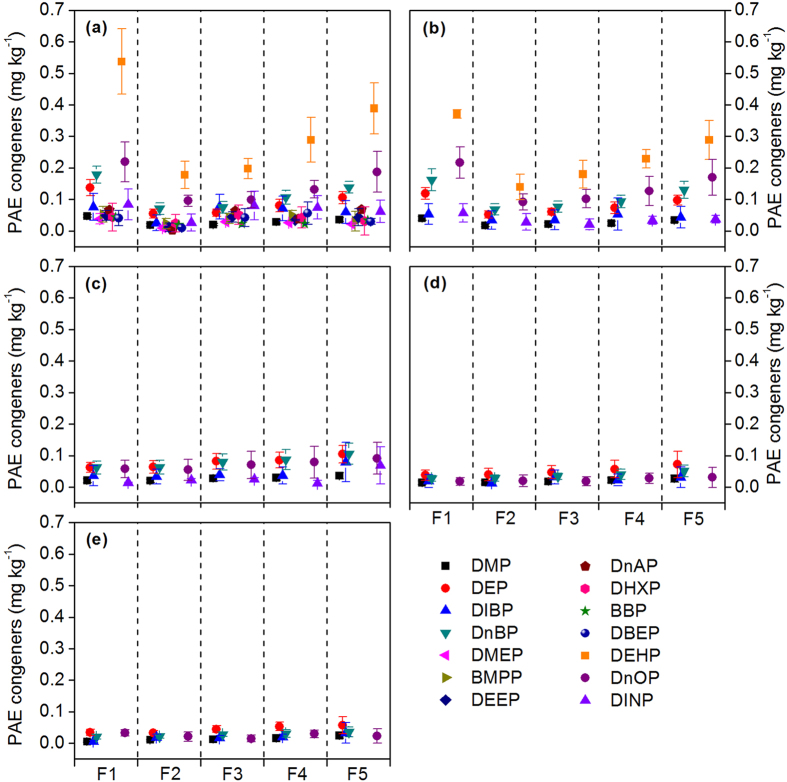
Concentrations of individual PAE congeners in soil particle-size fractions in different soil layers. (**a**) Soil layer (0–20 cm). (**b**) Soil layer (20–40 cm). (**c**) Soil layer (40–60 cm). (**d**) Soil layer (60–80 cm). (**e**) Soil layer (80–100 cm). In the x axis, F1, F2, F3, F4, and F5 represent soil coarse sand (250–2000 μm), fine sand (53–250 μm), coarse silt (20–53 μm), fine silt (2–20 μm), and clay (<2 μm), respectively.

The concentrations of ΣPAE, six priority PAEs, and individual PAE congeners showed various distribution patterns in different soil particle-size fractions. In soil layers at 0–20 and 20–40 cm depth, coarse sand (250–2000 μm) showed the highest values for all the concentrations of ΣPAE, six priority PAEs, and individual PAE congeners, followed by clay (<2 μm), fine silt (2–20 μm), coarse silt (20–53 μm), and fine sand (53–250 μm), respectively (Figs 1a,b and 2), which is consistent with the organic carbon distribution pattern (Fig. 1d and Supplementary Fig. S2a,b). Relative to other soil layers, the soils at 0–20 and 20–40 cm depths showed higher organic carbon content, and the PAE distribution pattern in different soil particle-size fractions was predominantly determined by the organic-matter adsorption^7^,^36^. In soil layers at 40–60, 60–80, 80–100 cm depths, clay (<2 μm) showed the highest concentrations of ΣPAE, six priority PAEs, and individual PAE congeners, followed by fine silt (2–20 μm), coarse silt (20–53 μm), fine sand (53–250 μm), and coarse sand (250–2000 μm), respectively (Figs 1a,b and 2), which shows a distribution pattern that is correlated to clay minerals content (Fig. 1e and Supplementary Fig. S2c,d). This observation indicates that, under low organic matter content, PAE distribution pattern in different soil particle-size fractions was most likely controlled by the clay-mineral adsorption^40^. Regarding the percentages of priority PAEs in ΣPAE, significant variation among different soil particle-size fractions was only found for the soil layer at 0–20 cm depth (Fig. 1c).

**Figure 3:**
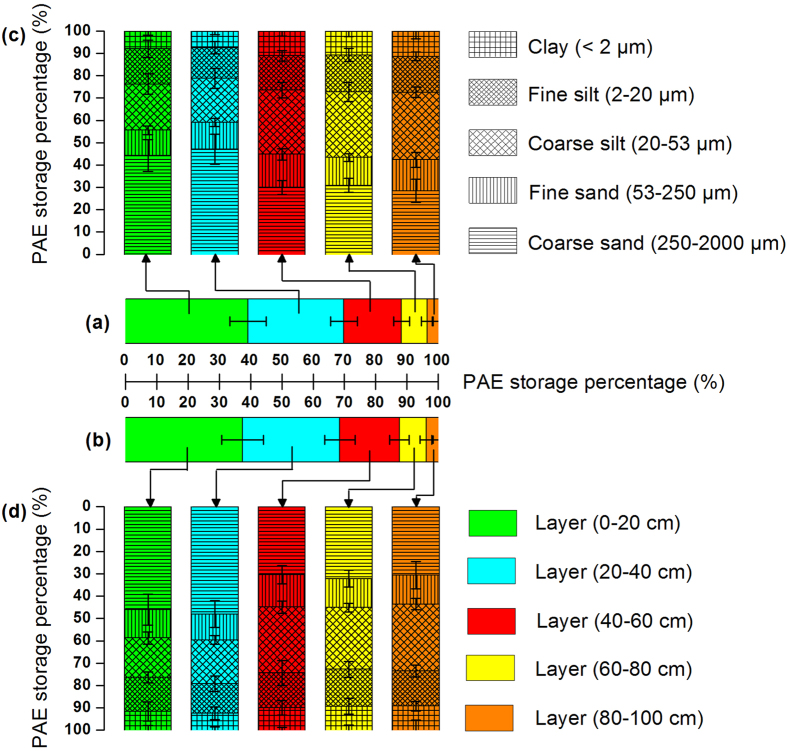
Distributions of ΣPAE and six priority PAEs storages in different soil layers and particle-size fractions. (**a**) Percentages of ΣPAE storages distributed in different soil layers. (**b**) Percentages of six priority PAEs storages distributed in different soil layers. (**c**) Percentages of ΣPAE distributed in different soil particle-size fractions in different soil layers. (**d**) Percentages of six priority PAEs distributed in different soil particle-size fractions in different soil layers.

**Figure 4:**
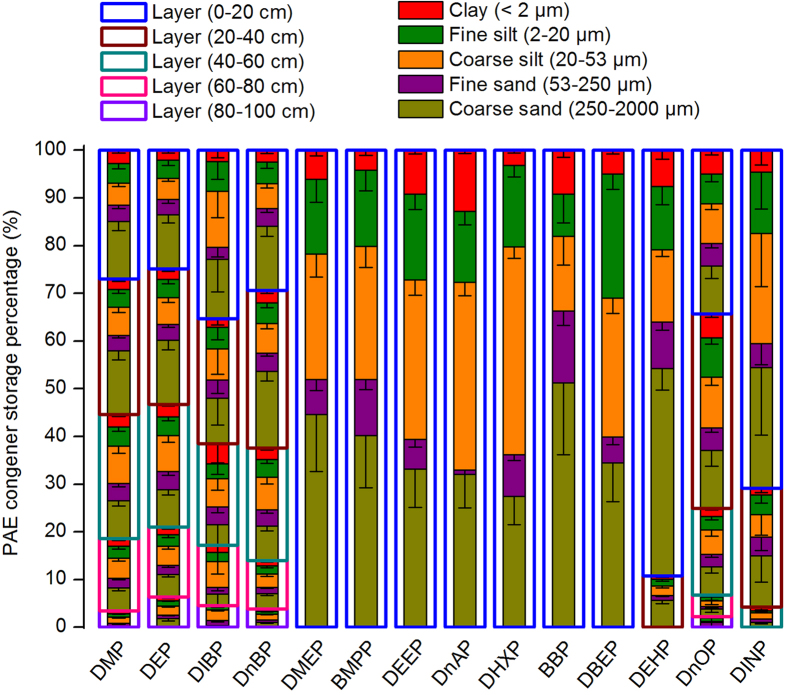
PAE congener storage distributions in different soil layers and particle-size fractions.

PAE congeners’ storages in different soil layers are presented in Supplementary Table S6. Among the 14 PAEs, DEHP and DnOP show the highest storage in soil because these two compounds had relatively higher inputs from wastewater (see Supplementary Table S5) and longer half-lives in the same soil condition (see Supplementary Table S7). Similar to the PAE concentration, PAE storage in soil layers gradually declined with depth (Figs 3a,b and 4). The total storage of ΣPAE and six priority PAEs in soil layers at 0–40 cm depth account for 70.1 ± 4.3% and 68.3 ± 5.9% of those in the entire soil profile, respectively. The distribution pattern of ΣPAE and six priority PAEs storages in different soil particle-size fractions varied in different soil layers (Fig. 3c,d), but the highest percentages of PAE storages were always found either in the coarse sand or in the coarse silt, and the clay fractions constantly showed the lowest PAE storages. This observation was also applicable to the individual PAE congeners (Fig. 4).

### Occurrences of PAEs in maize and wheat tissues

PAE concentration and detection frequencies in different tissue have never been reported and were investigated in this study in samples of maize and wheat for the first time. A summary of the data is presented in Supplementary Tables S8 and S9. Among the 14 PAEs, DnOP, DEHP, DnBP, and diisobutyl phthalate (DIBP) exhibited higher concentrations in plant tissues than the other PAE congeners, which may be related to their higher storages in soils. A significant difference in plant tissues was observed for concentrations of ΣPAE, six priority PAEs, and individual PAE congeners (Supplementary Fig. S3a,b and Table S8); and grains showed the highest concentrations of ΣPAE and six priority PAEs: 21.1 ± 7.1 and 11.0 ± 5.1 mg kg^−1^ in maize, as well as 16.2 ± 3.4 and 8.0 ± 2.6 mg kg^−1^ in wheat. Roots had the second highest concentrations: 14.9 ± 3.9 and 7.8 ± 1.9 mg kg^−1^ in maize, as well as 4.9 ± 1.4 and 3.1 ± 1.1 mg kg^−1^ in wheat. The lowest concentration was in shoot: 5.8 ± 2.9 and 3.6 ± 1.4 mg kg^−1^ in maize, as well as 2.2 ± 0.6 and 1.5 ± 0.4 mg kg^−1^ in wheat. Similar to ΣPAE and six priority PAEs, the majority of PAE congeners (except DMP, DEP, DIBP, and DnBP in maize and bis(4-methyl-2-pentyl) phthalate (BMPP) in wheat) were detected with highest concentrations in grain and lowest concentrations in shoot. The results indicate that PAEs are more easily accumulated in maize and wheat grains than in other tissues. The higher accumulation of chemicals in aboveground organs than in underground organs was also reported by others^41^,^42^,^43^. Felizeter *et al*.^43^ studied the uptake of perfluorobutanoic acid and perfluoropentanoic acid by lettuce (*Lactuca sativa*) and pointed out that chemicals easily passed through (or bypassed) the Casparian strip and accumulated in the vascular tissue in connection with the leaf and grain. This means that some chemicals can be absorbed through the active transport by plant root and subsequently translocated with transpiration streams within xylem^44^. Considering that maize and wheat are vascular plants with great transpiration rates, we speculate that the same mechanisms account for the higher accumulation of PAE in grains and leaves. When grains develop in the heading stages, organics would be allocated from the source organ leaves to storage organ grains^45^, which probably further promotes the accumulation of PAEs in grains through the cotransport between organics (e.g. sugars and amino acid) and PAEs. In addition, adsorption and subsequent absorption of PAEs evaporated from contaminated soils by leaf stomas can also partly explain why more PAEs were accumulated in leaves than in shoots. Nevertheless, additional studies are required to explore this further and evaluate potential differences of PAE concentrations between source and storage organs in wastewater irrigated maize and wheat crops to validate impacts on PAE transport.

In comparison with wheat, the maize had higher concentrations of ΣPAE, six priority PAEs, and individual PAE congeners in plant tissues (Supplementary Fig. S3a,b and Table S8), which is most likely due to the higher proportion of maize root weight, length, and surface area distributed in the upper soil profile with higher PAE storage (Supplementary Fig. S4). Moreover, the percentage of the six priority PAEs in ΣPAE in the tissues of maize and wheat significantly varied (Supplementary Fig. S3c). The highest percentages were in the shoots of both maize and wheat (63.5 ± 7.8% and 68.0 ± 6.7%), whereas the lowest percentages were in grains (50.6 ± 7.7% and 49.0 ± 7.3%). Except for the grain, the percentages of the six priority PAEs in ΣPAE in wheat tissues were higher than the ones in maize tissues (Supplementary Fig. S3c).

**Figure 5:**
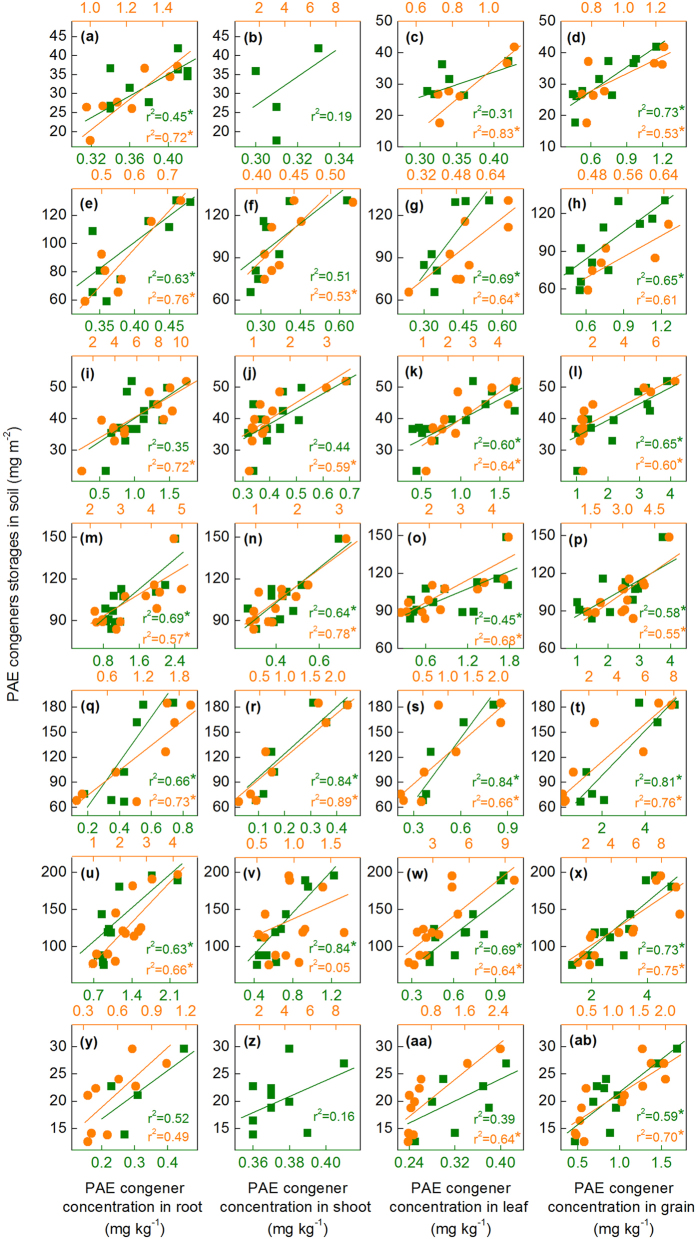
Correlations between PAE congener concentrations in plant tissues and PAE congener storages in soils. (**a**)–(**d**) DMP. (**e**)–(**h**) DEP. (**i**)–(**l**) DIBP. (**m**)–(**p**) DnBP. (**q**)–(**t**) DEHP. (**u**)–(**x**) DnOP. (**y**)–(**ab**) DINP. Circles and squares denote maize and wheat, respectively. An asterisk (*) indicates statistically significant (*P* < 0.05).

**Figure 6:**
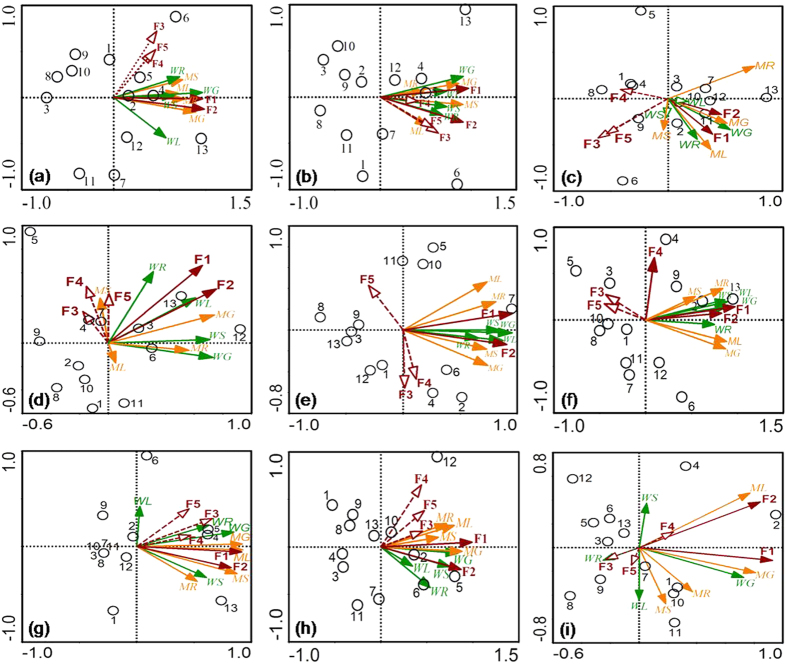
Redundancy analysis ordination diagram for concentrations of ΣPAE (**a**), the six priority PAEs (**b**), DMP (**c**), DEP (**d**), DIBP (**e**), DnBP (**f**), DEHP (**g**), DnOP (**h**), and DINP (**i**) associated with their corresponding PAE storage in different soil particle-size fractions. MR, MS, ML, and MG represent maize root, shoot, leaf, and grain, respectively; WR, WS, WL, and WG represent wheat root, shoot, leaf, and grain, respectively; F1, F2, F3, F4, and F5 represent coarse sand (250–2000 μm), fine sand (53–250 μm), coarse silt (20–53 μm), fine silt (2–20 μm) and clay (<2 μm), respectively. Major soil fractions are indicated by solid lines with filled arrows while minor soil fractions are shown using dotted lines with unfilled arrows. Samples are represented by open circles.

The relationships between the concentrations of ΣPAE, six priority PAEs, and PAE congeners (including DMP, DEP, DIBP, DnBP, DEHP, DnOP, and di-iso-nonyl phthalate (DINP) with relatively higher detection frequencies in soils and crops) in plant tissues and their corresponding storages in soils, were analyzed and summarized in Supplementary Fig. S5 and Fig. 5. The results showed that PAE concentrations in maize and wheat tissues correlated well with the PAE storage in soils. Organic pollutants associated with different soil particle-size fractions result in different bioavailability for plants^31^,^46^. Accordingly, redundancy analysis was performed to unravel the contributions of PAE storage in different soil particle-size fractions to PAE concentrations in maize and wheat tissues. Results showed that the concentrations of ΣPAE, six priority PAEs, and individual PAE congeners in maize and wheat tissues were significantly influenced by PAE storage in soil coarse sand (250–2000 μm) and fine sand (53–250 μm) rather than other fractions (Fig. 6). Based on the 5% level in partial Monte Carlo permutation test, coarse sand (250–2000 μm) and fine sand (53–250 μm) solely explained 37.4% and 36.0% of the variation of ΣPAE, 36.8% and 32.8% of the variation of six priority PAEs. Furthermore, coarse sand and fine sand explained 34.5%~46.5% and 26.5%~41.6% of the variation of seven individual PAE congeners (Supplementary Table S10). The weight proportion distributed in coarse sand (250–2000 μm) and fine sand (53–250 μm) fractions was not significantly higher than that distributed in other soil fractions (Supplementary Table S11). Moreover, Figs 3 and 4 showed that all the percentages of ΣPAE, six priority PAEs, and individual PAE congeners distributed in coarse sand (250–2000 μm) and fine sand (53–250 μm) fractions were also not significantly higher than those distributed in other soil fractions, especially in the deeper soil where PAEs might greatly contribute to the plant PAE levels. These results indicate that the PAEs in plant tissues were more related to the “particle-size” of fractions rather than the PAE storage percentage in soil fractions. As depicted in Fig. 1d, the coarse sand (250–2000 μm) and fine sand (53–250 μm) fractions have the highest and the lowest organic carbon content, respectively, whereas they both exhibit a high level of active organic matter (see the columns filled with oblique grids in Fig. 1d). The active organic matters are mostly comprised of slightly decomposed plant debris and partially degraded residues, being favorable to the adsorption of PAEs^47^. For the soil fractions with smaller particle size (e.g. soil fractions of coarse silt, fine silt, and clay), the lower active organic matter content is explained not only by the sieve-exclusion of plant debris in these fractions, but also by the stronger lock effect of organic matters by the smaller mineral particles. Meanwhile, coarse sand and fine sand show higher soil enzyme activity than other fractions (Fig. 1f), which was associated with the presence of higher levels of active organic matter in these fractions. In turn, the higher soil enzyme activity means that the active organic matter was readily degraded by microorganisms resulting in a faster updating rate (or a shorter turnover time) for the active organic matters. As a result, a higher flux of active organic matter facilitated the release of the adsorbed PAEs and their subsequent absorption by plants.

**Figure 7:**
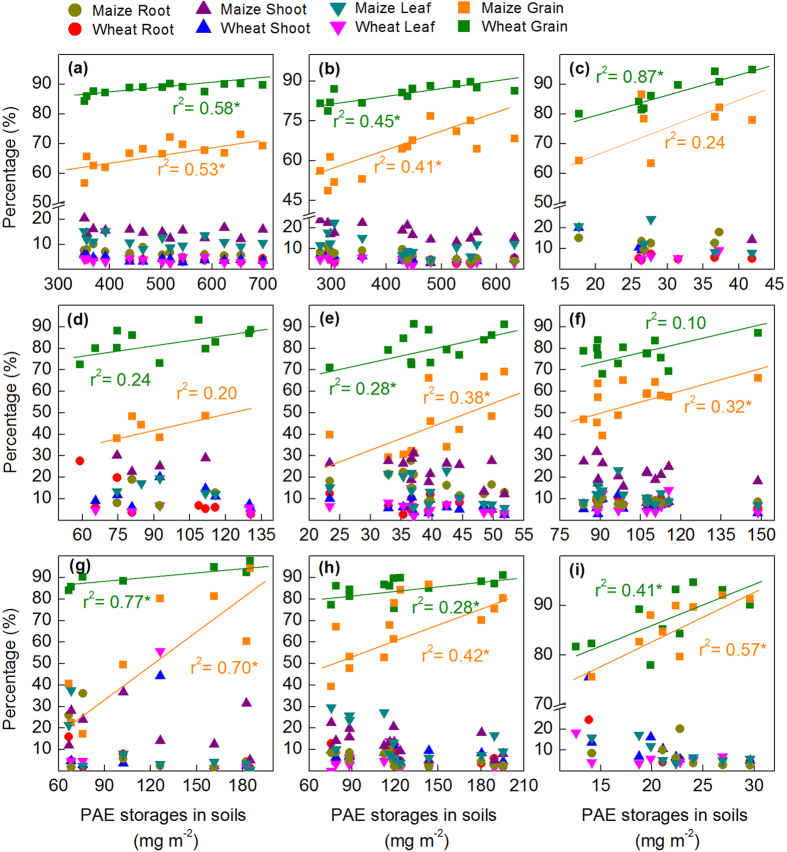
Relationship between percentage of PAE storage in different plant tissues and PAE storage in soil for (**a**) ΣPAE. (**b**) The six priority PAEs. (**c**) DMP. (**d**) DEP. (**e**) DIBP. (**f**) DnBP. (**g**) DEHP. (**h**) DnOP. (**i**) DINP. An asterisk (*) indicates statistically significant (*P* < 0.05).

The storages of ΣPAE, six priority PAEs, and individual PAE congeners varied greatly in different plant tissues (Supplementary Fig. S6). The grains had the highest storage values for ΣPAE, six priority PAEs, and individual PAE congeners, accounting for 66.7 ± 4.4%, 63.4 ± 8.8%, and 44.4 ± 13.9–100%, respectively, in maize, as well as 88.3 ± 1.8%, 85.3 ± 3.4%, and 70.6 ± 1.7–100%, respectively, in wheat. The high PAE storage in grains was largely attributed to the higher biomass in grains than that in other plant tissues (Supplementary Table S12). It is noted that, with increased PAE storage in soil, although the PAE storage in different plant tissues gradually increased, only the PAE proportions distributed in cereal grains increased significantly, such a trend was applicable for ΣPAE, six priority PAEs, and individual PAE congeners (Fig. 7). This finding implies that wastewater irrigation has a positive effect on crop yield because it can improve soil quality and increase nutrient content in soil to a certain extent^25^, but also leads to PAE allocation in cereal grains and thus poses a major threat to food resources. Future population growth and water scarcity will generate significant risks to global food security and thus drive the need to reuse wastewater for agricultural irrigation^48^. Therefore, it is a great challenge to study and optimize the wastewater management system to mitigate the contradiction between food security associated with PAE’s and other CECs pollution and water scarcity in the future.

### Bioconcentration factor (BCF) and translocation of PAE congeners

**Figure 8:**
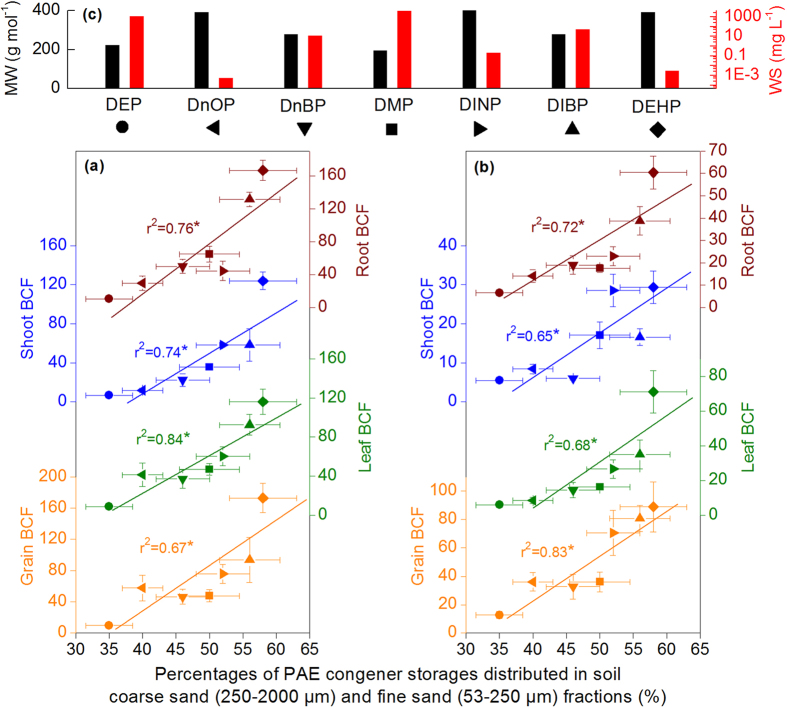
Relationships of BCFs of PAE congeners for maize (**a**) and wheat (**b**) tissues with percentages of PAE congeners storages distributed in the soil coarse sand (250–2000 μm) and fine sand (53–250 μm) fractions. (**c**) The legends, molecular weights (black column), and water solubility (red column) for various PAE congeners involved in (**a**,**b**). An asterisk (*) indicates statistically significant (*P* < 0.05).

BCF is often expressed by the ratio of plant PAE concentration to surface soil PAE concentration^9^,^17^,^18^. This ratio may be appropriate for vegetables with roots distributed in surface soil layers. However, roots of maize and wheat can deeply penetrate into soil profile and take up PAEs simultaneously on the surface and in underlying soil layers^49^. As a result, BCF calculated based on surface soil PAE concentration could not objectively reflect PAE accumulation capacity of maize and wheat. In addition, considering that various PAE compounds present different distribution patterns along soil depth, BCF calculated based on surface soil PAE concentration is inapplicable to compare the enrichments of different PAE compounds in plants. Therefore, this study defines for the first time BCF as the ratio between pollutant concentration in plant tissues and average PAE concentration in a thicker soil layer (0 cm to 100 cm). PAE congeners with greater molecular weight (MW) have lower water solubility (WS), which may make them vulnerable to soil adsorption but hardly absorbed by plants^50^,^51^. This study also analyzes the BCFs of DMP, DEP, DIBP, DnBP, DEHP, DnOP, and DINP, which have relatively higher detection frequencies in soils and crops (Supplementary Tables S3 and S9). The BCFs of PAE congeners in both maize and wheat tissues are determined to poorly correlate with their MW and WS but correlate well with their proportions distributed in coarse sand (250–2000 μm) and fine sand (53–250 μm) fractions with higher bioavailability (Fig. 8). This finding evidences that the enrichment capacity of plant to soil PAE congeners is not completely determined by PAE molecular structures but is largely subject to PAE distribution pattern in soil particle-size fractions. For maize and wheat tissues, especially grains, their BCFs with respect to PAE congeners are far greater than one, indicating the strong PAE enrichment abilities of maize and wheat. The BCFs of PAE congeners in both whole maize and wheat plants are equal to those of herbaceous plants^52^, but obviously higher than those of vegetables in previous studies^8^,^17^,^18^,^20^, implying the higher PAE enrichment ability of maize and wheat than vegetables.

The translocation of compounds from root to shoot and subsequently to leaf and grain can be described by the chemical’s concentration ratios in different plant tissues^43^. In this study, PAE concentration ratios in different plant tissues were calculated for PAE congeners with relatively higher detection frequencies in maize and wheat tissues, as shown in Supplementary Fig. S7. PAE concentration ratios of shoot to root in both maize and wheat decreased with increasing molecular weight from DMP to DIBP, then increased gradually from DnBP to DINP. Conversely, PAE concentration ratios of leaf to shoot and grain to shoot in both maize and wheat first increased and then decreased with increasing molecular weight. These results are not consistent with the general assumption associated with the translocation of chemicals within plants, namely that the sorption of hydrophobic organic molecules (e.g. the one with larger molecular weight) by plant tissues may decrease their distribution in the organs farther away from the root apex^43^. This inconsistency suggests that the link between the translocation of PAEs in crops and the PAE molecular weight was weak, and the translocation is a complex process governed by a combination of soil environmental effects, plant species, and chemical type. More studies are needed to explore the exact translocation routes of PAEs in these two cereal crops.

### Health risks of PAE Congeners in maize and wheat grains

**Figure 9:**
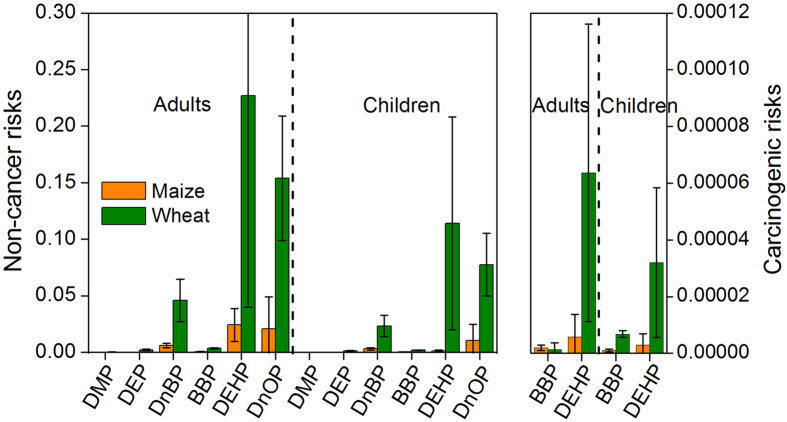
Health risks of PAE congener to adults and children via dietary intake of maize and wheat grains.

In this investigation the risks of PAEs (including DMP, DEP, DnBP, BBP, DEHP, and DnOP) in cereals were evaluated for the first time; results of risk assessment are summarized in Fig. 9. The non-cancer risks of the six priority PAE congeners to adult and children were within the suggested tolerance (*HQ* < 1), indicating that there would be negligible non-cancer risks associated with exposure to PAE congeners by dietary intake of maize and wheat from the wastewater irrigated area of Wangyanggou. For the six priority PAE congeners, DEHP in both maize and wheat grains had the highest non-cancer risks for adults and children, which is consistent with previous studies in regard to vegetables and other plants^8^,^9^,^53^. The non-cancer risk of DnOP was the second highest and DMP had the lowest non-cancer risk. The carcinogenic risks of BBP and DEHP to adults and children via dietary intake of maize and wheat were higher than 1 × 10^−6^. This result reflects that these two PAE congeners could pose potential carcinogenic risks to human health. The non-cancer and carcinogenic risks of the six priority PAE congeners in wheat were higher than those of maize, indicating that the effect of PAEs on wheat food safety should be prioritized. The non-cancer risks of five priority PAE congeners (except DEHP) and the carcinogenic risk of BBP in wheat from the wastewater irrigated area of Wangyanggou were greater than those in previously studies on vegetables^8^, but non-cancer risks of the six priority PAE congeners and carcinogenic risks of BBP and DEHP in maize from the wastewater irrigated area of Wangyanggou were lower. Almost all of the non-cancer and carcinogenic risks to humans for PAEs were due to direct intake of the edible parts^8^. PAE intake may pose a special health hazard to vulnerable populations such as pregnant women and children^4^,^54^. Even when the overall health risk of PAE is low in maize and wheat crop systems, the potential damage of PAE to human health via long-term exposure to low doses may still deserve further attention.

## Conclusion

In conclusion, this study reports on new findings related to the occurrence, bioavailability and health risks of PAEs in a soil-cereal crop system. Our results revealed an important link between PAE presence and distribution in cereal crops and PAE distribution patterns in soil particle-size fractions. Due to the higher active organic carbon flux and enzyme activity, the soil coarse sand (250–2000 μm) and fine sand (53–250 μm) fractions showed the greatest PAE uptake and BCFs in maize and wheat tissues. The risk of PAE exposure via dietary intake of maize and wheat grains to human health should be further investigated, considering that the BCFs of PAE congeners in both whole maize and wheat grains were higher than those in vegetables reported in previous studies. Our observations suggest a need for better management of wastewater irrigation due to the fact that more PAEs are detected in cereal grains with increasing wastewater irrigation. Overall, these novel findings provide basic information for formulating national standards on PAE’s pollution control in agricultural settings.

## Materials and Methods

### Study area and sampling

The study area is located near the city of Shijiazhuang in Hebei Province, Northern China (Supplementary Fig. S8). Farmlands in this area have been irrigated with wastewater for more than 30 years because of the high demand and shortage of freshwater. Summer maize (*Zea mays* L.)-winter wheat (*Triticum aestivum* L.) rotation (covering up to 60% of the arable land) is the predominant crop system in this area. Irrigation was applied four times during growing seasons of maize and three times during growing seasons of wheat (Supplementary Table S13).

Mature maize and wheat plant samples from 13 sites were collected in October 2012 and June 2013, respectively. At each site, above-ground plant tissue samples were collected from four random plots to determine their yields. The area of each plot is 10 m^2^ for maize and 6 m^2^ for wheat. One random subplot (0.5 m × 0.5 m) in each plot of both maize and wheat was sampled up to a depth of 100 cm at 5 cm intervals to collect root samples for measuring biomass, length, and surface area. To determine the PAE concentrations in the plant tissues, five subsamples of plant tissues from maize or wheat in each plot were randomly selected and mixed into one sample. Roots, shoots, leaves, and grains from both maize and wheat samples were then rinsed with deionized water, freeze-dried, ground, and homogenized by sieving through #100 mesh. Soil sampling was conducted simultaneously when the maize and wheat plant samples were collected. One soil profile in each plot was randomly selected, and soils were sampled to a depth of 100 cm at 20 cm intervals. At each depth of soil profile, a sample was first collected with a ring sampler to determine the soil bulk density, and approximately 1 kg of soil was then collected for chemical analysis. The wastewater samples from 13 sites and ground water samples from 3 sites were also collected. All pretreated plant tissues and soil samples were stored at −20 °C and water samples were stored at 4 °C until analysis.

### Soil particle-size fractionation

All bulk soil samples passing through sieve #10 mesh (smaller than 2 mm particle size) were divided into coarse sand (250–2000 μm), fine sand (53–250 μm), coarse silt (20–53 μm), fine silt (2–20 μm), and clay (<2 μm) following the method described in Amelung *et al*. with some modifications^55^. Freeze-dried soil samples (500 g) were treated ultrasonically at 40 kHz in an ultrasonic bath for 30 min with a water to soil ratio of 10:1. Each fraction was collected by settling from a suspension at an appropriate sedimentation time as determined by Stokes’ Law. The different particle-size fractions were then freeze-dried and weighed to calculate their relative percentages. Finally, the bulk soil and soil fractions were ground for PAE extraction and chemical analysis. Note that the mineral composition was used to denote the soil fraction even though minerals were not the only content.

### PAE extraction and analysis

PAEs in plant tissues and soils were identified and quantified using methods that have been validated and described previously^8^. Each aliquot of 3 g of plant tissue sample (or 5 g of soil sample) was spiked with a surrogate standard mixture of di-n-butyl phthalate-d4 (DnBP-D4), diphenyl isophthalate (DPIP) and diphenyl phthalate (DPP) in order to test target PAE recoveries. The samples were extracted by sonication for 30 min with 20 mL each of acetone and hexane. The solvent extracts were rotary evaporated to near dryness. The concentrated extracts were then solvent-exchanged into hexane and cleaned on a column packed with combined anhydrous sodium sulfate and neutral silica gel. The water samples (1 L) were filtered at a flow rate of 5 mL min^−1^ by using solid-phase extraction (SPE) cartridges (HLB cartridges, 500 mg per 6 mL; Waters) pre-conditioned with 5 mL of dichloromethane, 5 mL of methanol, and 5 mL of ultra-pure water. The PAEs were eluted from the cartridge by using 10 mL of dichloromethane and collected in 10 mL glass tubes with stoppers. The eluate was evaporated under a stream of purified N_2_ until the final volume of 1 mL. PAEs were identified and quantified using 7890N gas chromatograph coupled with 5975C mass selective detector. Chromatographic separation was achieved on a DB-5MS capillary column (30 m length, 0.25 mm ID, and 0.25 μm film thickness). A splitless injector was used and held at 280 °C. The transfer line temperature was maintained at 280 °C, and the ion temperature was kept at 230 °C. The carrier gas was helium. The oven temperature range was 70 °C (2 min) to 130 °C (1 min) at 20 °C min^−1^, then increased to 240 °C (1 min) at 5 °C min^−1^, followed by 260 °C (1 min) at 5 °C min^−1^, then to 270 °C (1 min) at 5 °C min^−1^, and finally to 280 °C (7 min) at 10 °C min^−1^.

### Quality assurance and quality control (QA/QC)

During analysis, all data were subject to strict quality control and assurance measures as described previously^11^. Concentrations of all PAE congeners were quantified by the external standard method and the standard curves of 14 target PAE congeners were all at significant level (r^2^ > 0.999, *P* < 0.01). The spiking concentrations were at least three times the original matrix concentrations in plant tissues and soils. The recoveries of the 14 target PAE congeners were 82.4% to 110.2% in the spiked blank samples and 84.8% to 109.6% in the spiked plant tissue and soil samples. The PAE recoveries in the sum of particle-size fractions versus bulk soil in different soil layers were over 90% for most of PAE congeners (see Supplementary Table S14), indicating little loss of PAEs during the soil particle-size fractionation. All equipments were rinsed with acetone and hexane to avoid contamination. A laboratory blank sample was incorporated with every batch of 10 samples to check for interference and contamination. Each PAE concentration in the plant tissue and soil samples was blank corrected. The method detection limits for quantification of the 14 target PAEs were 1.34, 1.56, 2.10, 1.13, 1.43, 1.21, 1.07, 1.11, 1.94, 1.25, 0.13, 0.48, 0.69, and 0.81 μg kg^−1^ for DMP, DEP, DIBP, DnBP, DMEP, BMPP, DEEP, DnAP, DHXP, BBP, DBEP, DEHP, DnOP, and DINP, respectively, and the instrument detection limits of PAEs were calculated as three times the signal-to-noise ratio and varied from 1.61 μg kg^−1^ to 5.32 μg kg^−1^.

### PAE concentration and storage calculations

PAE concentrations in plant tissues and soils were normalized to dry plant tissue and soil weight, respectively. PAE storage in plant tissues, bulk soils, and soil particle-size fractions were calculated as follows:


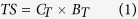



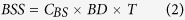



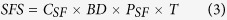


where *TS* is the PAE storage in plant tissues (mg m^−2^), *C*_*T*_ is the PAE concentration in plant tissues (mg kg^−1^), *B*_*T*_ is the plant tissue biomass per area (kg m^−2^), *BSS* is the PAE storage in bulk soil (mg m^−2^), *C*_*BS*_ is the PAE concentration in bulk soil, *BD* is the soil bulk density (kg m^−3^), *T* is the thickness of soil sublayer (m), *SFS* is the PAE storage in the soil particle-size fraction (mg m^−2^), *C*_*SF*_ is the PAE concentration in soil fraction (mg kg^−1^), and *P*_*SF*_ is the mass percentage of soil particle-size fraction (%). PAE storages in bulk soil and soil particle-size fractions in the entire soil layer (0 cm to 100 cm) were expressed as the sums of *BSS* and *SFS* in different soil sublayers, respectively.

### Physicochemical analyses of soils

For bulk density determination, soil samples were weighed after they were oven-dried at 105 °C. All bulk soil and soil fractions for total organic carbon (TOC) measurements were pretreated with excess 0.5 M hydrochloric acid to remove carbonates at room temperature, then rinsed, and freeze-dried. TOC contents were determined by an elemental analyzer (VARIO EL cube). The active organic carbon contents in bulk soil and soil fractions were determined using 60 mM KMnO_4_ as described by Vieira *et al*.^56^. Enzymatic activities in bulk soil and soil fractions were expressed as the total activities of dehydrogenase, urease, alkaline phosphatase, β-glucosidase, and arylsulphatase, which were determined using the methods of Liang *et al*.^57^. Semi-quantitative measurements of clay minerals in bulk soils and soil fractions were performed by X-ray diffraction as described by Zanelli *et al*.^58^.

### Determinations of plant tissue yield, root length and root surface area

In each plot, roots, shoots, leaves, and grains from both maize and wheat plants were separated, and their yields were determined by weighing after they were oven-dried at 60 °C. Separated root fractions from soil were scanned (Epson V700, Indonesia) and then analyzed by WinRHIZO version 5.0 (Régent Instrument Inc., Québec, Canada). The total root length and surface area for each sample were calculated as described by Mosaddeghi *et al*.^59^.

### Health risk assessment

The potential health risks for exposure to PAEs were assessed using the methods recommended by USEPA^60^. Among the individual PAE congeners studied, DMP, DEP, DnBP, and DnOP were considered non-carcinogen harmful, whereas BBP and DEHP were considered to be potential carcinogens. The non-cancer effect of DMP, DEP, DnBP, BBP, DEHP, and DnOP via dietary intake of maize and wheat was expressed as a dimensionless hazard quotient (*HQ*), calculated as follows:


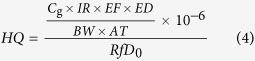


where *C*_*g*_ is the concentration of the target chemical in the plant grain (mg kg^−1^), *IR* is the daily intake rate of plant grain (mg day^−1^), *EF* is the exposure frequency (day year^−1^), *ED* is the exposure duration (years), *BW* is the body weight (kg), *AT* is the average lifetime exposure (days), and *RfD*_*o*_ is the health guideline value as reference dose for ingestion and intake of contaminated food (mg kg^−1^ day^−1^).

The carcinogenic risk (*CR*, dimensionless) of BBP and DEHP via dietary intake of maize and wheat was calculated by:


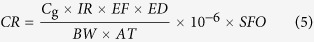


where *SFO* is the oral slope factor ((mg kg^−1^ day^−1^) ^−1^).

If the exposure level exceeds this threshold (i.e., *HQ* > 1), a potential adverse health effect exists. The estimated carcinogenic risk may be considered very low at *CR* value < 10^−6^. The parameter values (see Supplementary Table S15) for the non-cancer and carcinogenic risks assessments were based on the study of Wang *et al*.^8^.

### Statistical analysis

PAE concentrations in samples were represented as geometric mean ± geometric standard deviation. ANOVA was used to compare indices among different soil particle-size fractions and between maize and wheat tissues. Linear regression analysis was performed to assess the correlations between PAE concentrations in plant tissues and PAE storages in soils, between percentages of PAEs distributed in plant tissues and PAE storages in soils, and between BCFs in plant tissues and percentages of PAE congener storages distributed in coarse sand (250–2000 μm) and fine sand (53–250 μm). Both ANOVA and linear regressions were conducted with SPSS 18.0 (SPSS, Inc.), and the results were considered significant at *p* < 0.05. CANOCO 4.5 (Centre for Biometry, Wageningen, The Netherlands) was used to determinate the multivariate relationships between PAE concentrations in plant tissues and PAE storages in soil particle-size fractions. Detrended correspondence analysis (DCA) was performed first to choose between linear and unimodal response model for data of PAE concentrations in plant tissues. The length of the first DCA ordination axis was 0.156 for the concentration of ΣPAE (total of 14 PAEs), 0.216 for the total concentration of six priority PAEs, and 0.269–1.850 for the concentrations of individual PAE congeners, all of which were less than 3.000. Accordingly, redundancy analysis (RDA) was performed to ordinate the PAE concentrations in plant tissues to the PAE storages in soil particle-size fractions. Automated forward selection was used to determine which soil particle-size fractions significantly influenced PAE concentrations in plant tissues. Subsequently, variation partitioning was conducted to discriminate the influence of each significant particle-size fraction by using partial RDA. Monte Carlo-reduced model tests with 499 unrestricted permutations were performed to investigate the statistical significance at *p* < 0.05.

## Additional Information

**How to cite this article**: Tan, W. *et al*. Distribution patterns of phthalic acid esters in soil particle-size fractions determine biouptake in soil-cereal crop systems. *Sci. Rep.* **6**, 31987; doi: 10.1038/srep31987 (2016).

## Supplementary Material

Supplementary Information
