# Supplementary material for: Distribution patterns of phthalic acid esters in soil particle-size fractions determine biouptake in soil-cereal crop systems
Source: Sci Rep. 2016 Aug 24;6:31987. doi: 10.1038/srep31987 (PMC4995490; doi:10.1038/srep31987)
Supplement: Supplementary Information [file srep31987-s1.pdf]

Supplementary Information

Distribution patterns of phthalic acid esters in soil particle-size fractions determine biouptake in soil-cereal crop systems

Wenbing Tan[1,2,*], Yuan Zhang[3,4,*], Xiaosong He[1,2], Beidou Xi[1,2,5], Rutai Gao[1,2], Xuhui Mao[4], Caihong Huang[1,2], Hui Zhang[2], Dan Li[2], Qiong Liang[6], Dongyu Cui[2] & Akram N. Alshawabkeh[7]

[1]State Key Laboratory of Environmental Criteria and Risk Assessment, Chinese Research Academy of Environmental Sciences, Beijing 100012, China. [2]State Environmental Protection Key Laboratory of Simulation and Control of Groundwater Pollution, Chinese Research Academy of Environmental Sciences, Beijing 100012, China. [3]Hebei Provincial Academy of Environmental Sciences, Shijiazhuang 050037, China. [4]School of Resource and Environmental Science, Wuhan University, Wuhan 430079, China. [5]Lanzhou Jiaotong University, Lanzhou 730070, China. [6]College of Plant Science and Technology, Beijing University of Agriculture, Beijing 102206, China. [7]Civil and Environmental Engineering Department, Northeastern University, Boston, Massachusetts 02115, United States. *****These authors contributed equally to this work. Correspondence and requests for materials should be addressed to B.X. (email: xibeidou@yeah.net) or A.N.A. (email: aalsha@coe.neu.edu).

Chemicals and Standards. Fourteen PAEs (dimethyl phthalate (DMP), diethyl phthalate (DEP), diisobutyl phthalate (DIBP), di-n-butyl phthalate (DnBP), bis(2-methoxyethyl) phthalate (DMEP), bis(4-methyl-2-pentyl) phthalate (BMPP), bis(2-ethoxyethyl) phthalate (DEEP), di-n-pentyl phthalate (DnAP), di-n-hexyl phthalate (DHXP), butyl benzyl phthalate (BBP), bis(2-butoxyethyl) phthalate (DBEP), bis(2-ethylhexyl) phthalate (DEHP), di-n-octyl phthalate (DnOP), di-iso-nonyl phthalate (DINP)) were selected as target compounds. Fourteen PAE standard mixtures and a surrogate standard mixture of di-n-butyl phthalate-d4 (DnBP-D4), diphenyl isophthalate (DPIP), and diphenyl phthalate (DPP) were purchased from AccuStandard, Inc. (New Haven, CT, USA). Water was triple-distilled using a Milli-Q purification system (Millipore, USA). All solvents were of pesticide residue grade and were obtained from OmniSolv (EM Science, Lawrence, KS, USA). Anhydrous sodium sulfate (Na_2SO_4), silica gel (60-100 mesh size), and alumina (neutral, 150 mesh size) were purchased from Sigma-Aldrich (St. Louis, MO, USA).

Compound	Structure	CAS n.	MW	Log Kow	Vapor Pressure (at 25 ℃)
Dimethyl phthalate	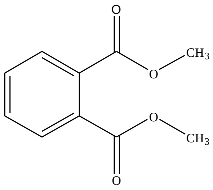	131-11-3	194.19	1.66	$3.08x10^{-3}$ mm Hg
Diethyl phthalate	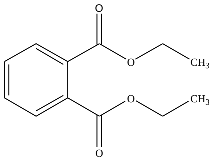	84-66-2	222.24	2.65	$2.10x10^{-3}$ mm Hg
Diisobutyl phthalate	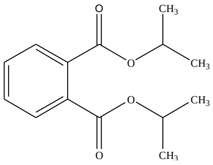	84-69-5	278.35	4.46	$2.41x10^{-3}$ mm Hg
Di-n-butyl phthalate	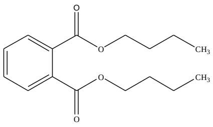	84-7-2	278.35	4.61	$2.28x10^{-4}$ mm Hg
Bis(2-methoxyethyl) phthalate	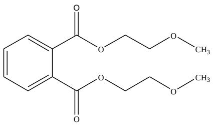	117-82-8	282.30	1.11	$2.28x10^{-4}$ mm Hg
Bis(4-methyl-2-pentyl) phthalate	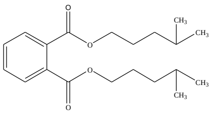	146-50-9	334.46	6.43	$4.25x10^{-5}$ mm Hg
Bis(2-ethoxyethyl) phthalate	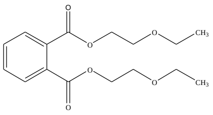	605-54-9	310.35	2.10	$1.44x10^{-4}$ mm Hg
Di-n-pentyl phthalate	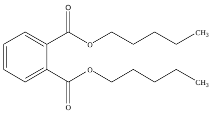	131-18-0	306.41	5.59	$1.96x10^{-4}$ mm Hg
Di-n-hexyl phthalate	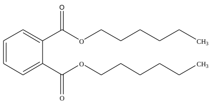	84-75-3	334.46	6.57	$1.97x10^{-5}$ mmHg
Butyl benzyl phthalate	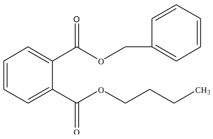	85-68-7	312.37	4.84	$4.4x10^{-5}$ mm Hg
Bis(2-butoxyethyl) phthalate	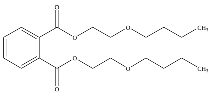	117-83-9	366.46	4.06	$2.17x10^{-3}$ mm Hg
Bis(2-ethylhexyl) phthalate	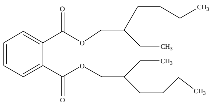	117-81-7	390.57	8.39	$2.03x10^{-5}$ mm Hg

Di-n-octyl phthalate	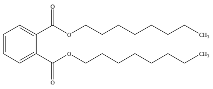	117-84-0	390.57	8.54	1.45x10^{-6} mm Hg
Di-iso-nonyl phthalate	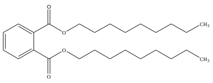	68515-48-0	418.62	9.52	3.88x10^{-7} mm Hg

Supplementary Table S1. Physico-chemical properties of the selected PAEs.

	0-20 cm	20-40 cm	40-60 cm	60-100 cm	80-100 cm
DMP	0.033±0.004	0.029±0.004	0.026±0.007	0.018±0.007	0.013±0.004
DEP	0.095±0.015	0.086±0.009	0.078±0.020	0.048±0.023	0.045±0.010
DIBP	0.052±0.007	0.042±0.007	0.029±0.007	0.013±0.004	0.011±0.003
DnBP	0.125±0.014	0.111±0.020	0.078±0.028	0.034±0.011	0.027±0.007
DMEP	0.021±0.012	ND	ND	ND	ND
BMPP	0.029±0.003	ND	ND	ND	ND
DEEP	0.029±0.018	ND	ND	ND	ND
DnAP	0.045±0.009	ND	ND	ND	ND
DHXP	0.025±0.015	ND	ND	ND	ND
BBP	0.026±0.009	ND	ND	ND	ND
DBEP	0.028±0.004	ND	ND	ND	ND
DEHP	0.364±0.058	0.280±0.020	ND	ND	ND
DnOP	0.181±0.052	0.161±0.046	0.075±0.042	0.030±0.017	0.034±0.010
DINP	0.052±0.008	0.034±0.005	0.020±0.003	ND	ND

Supplementary Table S2. PAE concentrations in different layers of soils irrigated with wastewater (mg kg^{-1}). ND, not detected.

	0-20 cm	20-40 cm	40-60 cm	60-100 cm	80-100 cm
DMP	96	92	81	46	3
DEP	100	92	81	50	3
DIBP	100	100	100	96	13
DnBP	100	100	100	100	50
DMEP	46	35	16	0	0
BMPP	23	0	0	0	0
DEEP	5	0	0	0	0
DnAP	4	0	0	0	0
DHXP	4	0	0	0	0
BBP	12	0	0	0	0
DBEP	4	0	0	0	0
DEHP	62	23	0	0	0
DnOP	100	100	81	42	8
DINP	85	62	12	0	0

Supplementary Table S3. PAE detection frequencies in different layers of soils irrigated with wastewater (%).

	0-20 cm	20-40 cm	40-60 cm	60-100 cm	80-100 cm
DMP	0.008±0.006	0.007±0.004	ND	ND	ND
DEP	0.009±0.003	0.006±0.002	ND	ND	ND
DIBP	0.009±0.004	ND	ND	ND	ND
DnBP	0.011±0.004	0.005±0.003	ND	0.005±0.004	ND
DMEP	ND	ND	ND	ND	ND
BMPP	ND	ND	ND	ND	ND
DEEP	ND	ND	ND	ND	ND
DnAP	ND	ND	ND	ND	ND
DHXP	ND	ND	ND	ND	ND
BBP	ND	ND	ND	ND	ND
DBEP	ND	ND	ND	ND	ND
DEHP	0.019±0.007	0.013±0.008	ND	0.008±0.005	ND
DnOP	0.035±0.010	0.016±0.006	ND	ND	ND
DINP	0.011±0.002	0.004±0.004	ND	ND	ND

Supplementary Table S4. PAE concentrations in different layers of soils irrigated with groundwater (mg kg^{-1}). ND, not detected.

	Wastewater	Groundwater
DMP	2.292±0.697	0.117±0.053
DEP	2.089±0.180	0.140±0.012
DIBP	2.295±0.536	0.158±0.070
DnBP	2.397±0.463	0.154±0.052
DMEP	1.892±0.530	ND
BMPP	0.666±0.039	ND
DEEP	1.081±0.226	ND
DnAP	1.085±0.187	0.145±0.015
DHXP	0.760±0.222	ND
BBP	1.408±0.377	ND
DBEP	0.724±0.358	ND
DEHP	5.089±0.931	0.221±0.029
DnOP	2.571±0.279	0.165±0.022
DINP	1.475±0.102	0.133±0.011

Supplementary Table S5. PAE concentrations in the wastewater and groundwater used for irrigation (μg L^{-1}). ND, not detected.

	0-20 cm	20-40 cm	40-60 cm	60-100 cm	80-100 cm
DMP	8.68±1.44	8.33±1.03	8.20±2.29	5.28±2.56	4.00±1.18
DEP	25.3±2.97	24.8±7.66	23.7±4.72	15.2±7.84	14.2±3.84
DIBP	12.9±2.11	12.4±2.40	9.27±2.58	3.76±1.71	3.38±1.02
DnBP	32.6±5.28	31.1±3.41	24.1±7.68	10.6±3.68	8.39±2.51
DMEP	4.83±3.29	-	-	-	-
BMPP	6.98±1.00	-	-	-	-
DEEP	7.13±5.36	-	-	-	-
DnAP	12.0±1.91	-	-	-	-
DHXP	5.32±2.81	-	-	-	-
BBP	6.39±2.14	-	-	-	-
DBEP	6.73±0.44	-	-	-	-
DEHP	89.6±19.7	83.8±11.4	-	-	-
DnOP	47.3±12.6	44.8±12.1	23.4±13.0	9.50±5.56	10.5±3.57
DINP	12.8±2.14	8.92±3.62	6.47±0.74	-	-

Supplementary Table S6. PAE storages in different layers of soils irrigated with wastewater (mg m^{-2}).

	Half-life (d)	Soil type	Reference
DMP	1.70[a]	Garden soil	Shanker et al.[1], 1985
	1.93[a]	Unknown	Russell et al.[2], 1985
	2.30[a]	Fluvo-aquic soil	Wang et al.[3], 2004
	12.5–41.7[b]	Unknown	Cousins et al.[4], 2003
DEP	1.83[a]	Unknown	Russell et al.[2], 1985
	3.70[a]	Fluvo-aquic soil	Wang et al.[3], 2004
	12.5–41.7[b]	Unknown	Cousins et al.[4], 2003
DnBP	0.43[a]	Unknown	Russell et al.[2], 1985
	1.80[a]	Garden soil	Shanker et al.[1], 1985
	2.80[a]	Sandy clay loam soil	Chang et al.[5], 2009
	6.70–15.8[a]	Silt soil	Chen et al.[6], 1997
	7.80[a]	Black soil	Xu et al.[7], 2008
	8.30[a]	Fluvo-aquic soil	Xu et al.[7], 2008
	8.50[a]	Fluvo-aquic soil	Wang et al.[3], 2004
	19.3[a]	Fluvo-aquic soil	Wang et al.[8], 1997
	41.7–125[b]	Unknown	Cousins et al.[4], 2003
BBP	41.7–125[b]	Unknown	Cousins et al.[4], 2003
DEHP	2.00-69.3[a]	Sandy loam soil	Rüdel et al.[9], 1993
	5.00–23.0[a]	Unknown	Borgert et al.[10], 1995
	6.30[a]	Sandy clay loam soil	Chang et al.[5], 2009
	17.3–46.2[a]	Silt soil	Chen et al.[6], 1997
	26.3[a]	Black soil	Xu et al.[7], 2008
	30.8[a]	Fluvo-aquic soil	Xu et al.[7], 2008
	53.3[a]	Sand soil	Shanker et al.[1], 1985
	58.0–147[a]	Sandy loam soil	Roslev et al.[11], 1998
	55.0–301[a]	Sandy loam soil	Madsen et al.[12], 1999
	125–416[b]	Unknown	Cousins et al.[4], 2003
DnOP	28.4[a]	Fluvo-aquic soil	Wang et al.[3], 2004

Supplementary Table S7. Summary of individual PAE congener biodegradation rates in soils. a, half-life determined by first-order kinetic equation fitting method. b, half-life determined by EQuilibrium Criterion (EQC) model.

	Maize root	Maize shoot	Maize leaf	Maize grain	Wheat root	Wheat shoot	Wheat leaf	Wheat grain
DMP	1.206±0.191	0.970±0.189	0.885±0.187	0.958±0.227	0.376±0.036	0.313±0.013	0.347±0.040	0.722±0.246
DEP	0.566±0.099	0.454±0.073	0.524±0.149	0.538±0.077	0.398±0.055	0.359±0.120	0.393±0.089	0.778±0.257
DIBP	6.028±2.790	1.512±0.812	2.388±1.183	2.475±2.078	1.011±0.266	0.435±0.099	0.943±0.475	2.154±1.059
DnBP	3.401±0.993	1.508±0.650	2.500±1.167	3.099±1.018	1.305±0.528	0.422±0.104	0.982±0.535	2.168±0.883
DMEP	0.040±0.038	ND	ND	0.134±0.069	0.111±0.032	ND	0.086±0.031	0.111±0.058
BMPP	0.685±0.128	ND	ND	1.188±0.508	0.059±0.033	ND	ND	0.021±0.002
DEEP	ND	ND	ND	0.690±0.132	ND	ND	ND	ND
DnAP	0.573±0.096	1.271±0.821	2.870±2.102	2.484±1.387	0.680±0.531	ND	0.357±0.027	1.849±0.867
DHXP	ND	ND	ND	0.478±0.107	0.432±0.092	ND	ND	0.509±0.111
BBP	0.430±0.011	ND	ND	0.528±0.254	ND	ND	ND	0.335±0.058
DBEP	1.057±0.411	0.590±0.114	1.887±1.406	3.383±1.014	0.482±0.159	ND	1.635±1.103	2.962±0.787
DEHP	1.007±0.789	0.755±0.690	0.627±0.710	2.072±2.853	0.415±0.169	0.233±0.108	0.491±0.183	2.150±1.770
DnOP	2.270±0.863	0.947±0.337	3.378±2.329	4.866±2.937	1.124±0.461	0.696±0.251	0.657±0.196	2.912±1.036
DINP	0.567±0.237	0.540±0.127	0.707±0.739	1.039±0.677	0.315±0.096	0.375±0.016	0.330±0.058	0.952±0.362

Supplementary Table S8. PAE concentrations in maize and wheat tissues (mg kg^{-1}). ND, not detected.

	Maize root	Maize shoot	Maize leaf	Maize grain	Wheat root	Wheat shoot	Wheat leaf	Wheat root
DMP	54	8	46	62	77	31	46	77
DEP	54	62	62	46	69	62	54	85
DIBP	100	100	100	100	100	100	100	100
DnBP	100	100	100	100	92	92	100	100
DMEP	0	0	0	0	0	0	8	0
BMPP	0	0	38	31	0	0	0	0
DEEP	0	0	0	0	0	0	8	0
DnAP	46	54	85	100	0	31	46	100
DHXP	23	8	0	0	69	0	0	38
BBP	23	8	0	15	31	0	0	0
DBEP	15	8	77	100	38	0	31	100
DEHP	100	100	100	100	77	8	77	77
DnOP	100	100	100	100	85	100	100	92
DINP	8	69	77	100	31	85	54	77

Supplementary Table S9. PAE detection frequencies in maize and wheat tissues (%).

PAE	Soil fraction	Eigenvalue	% Variation explains solely	*F* value	*P* value
ΣPAE	F1	0.374	37.4	12.69	0.002
	F2	0.360	36.0	13.84	0.004
	F3	0.098	9.8	1.879	0.146
	F4	0.088	8.8	1.468	0.204
	F5	0.079	7.9	1.659	0.220
The six priority PAEs	F1	0.368	36.8	12.47	0.002
	F2	0.328	32.8	9.589	0.002
	F3	0.096	9.6	3.436	0.062
	F4	0.136	13.6	1.295	0.260
	F5	0.073	7.3	2.693	0.102
DMP	F1	0.386	38.6	7.383	0.028
	F2	0.287	28.7	6.404	0.036
	F3	0.116	11.6	1.797	0.120
	F4	0.110	11.0	1.682	0.162
	F5	0.099	9.9	1.506	0.208
DEP	F1	0.396	39.6	1.833	0.025
	F2	0.405	40.5	1.991	0.013
	F3	0.054	5.4	0.706	0.769
	F4	0.089	8.9	1.229	0.280
	F5	0.056	5.6	0.743	0.666
DIBP	F1	0.465	46.5	16.13	0.002
	F2	0.416	41.6	12.56	0.002
	F3	0.062	6.2	0.437	0.692
	F4	0.030	3.0	0.383	0.758
	F5	0.026	2.6	0.958	0.350
DnBP	F1	0.364	36.4	21.72	0.002
	F2	0.265	26.5	10.41	0.006
	F3	0.077	7.7	1.801	0.174
	F4	0.207	20.7	6.680	0.006
	F5	0.086	8.6	2.066	0.168
DEHP	F1	0.345	34.5	16.18	0.004
	F2	0.281	28.1	10.37	0.004
	F3	0.125	12.5	4.101	0.052
	F4	0.158	15.8	5.069	0.050
	F5	0.091	9.1	2.052	0.148

DnOP	F1	0.398	39.8	15.50	0.002
	F2	0.313	31.3	9.370	0.002
	F3	0.092	9.2	1.714	0.174
	F4	0.097	9.7	1.840	0.188
	F5	0.099	9.9	1.873	0.160
DINP	F1	0.447	44.7	7.084	0.004
	F2	0.392	39.2	5.763	0.004
	F3	0.559	55.9	0.562	0.670
	F4	0.095	9.5	0.992	0.256
	F5	0.010	1.0	1.101	0.996

Supplementary Table S10. Eigenvalue, *F* and *P* values obtained from the partial RDAs testing the influence of the significant soil particle-size fractions on the PAE concentrations in plant tissues. Partial RDAs based on Monte Carlo permutation (n=499) kept only the significant soil particle-size fractions in the models. For each partial model, the other significant soil particle-size fractions were used as covariables. F1, F2, F3, F4, and F5 represent soil coarse sand (250–2000 μm), fine sand (53–250 μm), coarse silt (20–53 μm), fine silt (2–20 μm), and clay (<2 μm), respectively.

Coarse sand	Find sand	Coarse silt	Fine silt	Clay
24.9±3.1	18.7±2.6	25.1±2.9	17.0±2.7	6.4±1.2

Supplementary Table S11. The weight percentages of different soil particle-size fractions (%).

	Root	Shoot	Leaf	Grain
Maize	81.2±1.58	512±11.6	155±3.70	612±135
Wheat	78.0±3.36	179±3.85	86.3±3.16	540±74.7

Supplementary Table S12. Biomass of maize and wheat tissues (×10^{-3} kg m^{-2}).

	Irrigation	
	Date	Rate (mm)
Summer maize	15 Jun. 2012	60
	5 Jul. 2012	60
	5 Aug. 2012	30
	5 Sep. 2012	30
	Total	180
Winter wheat	20 Nov. 2012	60
	15 Apr. 2013	60
	15 May 2013	60
	Total	180

Supplementary Table S13. Wastewater irrigation information during the growing seasons of summer maize and winter wheat.

	0-20 cm	20-40 cm	40-60 cm	60-100 cm	80-100 cm
DMP	92.0±4.73	93.9±7.31	94.4±9.47	89.8±5.38	91.4±4.81
DEP	95.4±6.21	89.1±7.65	93.5±8.31	92.1±5.73	88.8±5.56
DIBP	107±16.3	94.6±7.94	102±15.5	105±11.5	114±21.9
DnBP	97.9±6.46	90.8±6.65	88.9±8.21	92.1±6.92	88.8±5.36
DMEP	109±9.82	-	-	-	-
BMPP	106±10.4	-	-	-	-
DEEP	118±19.4	-	-	-	-
DnAP	98.2±4.91	-	-	-	-
DHXP	101±5.17	-	-	-	-
BBP	85.8±3.41	-	-	-	-
DBEP	103±12.1	-	-	-	-
DEHP	92.3±5.52	94.9±4.59	-	-	-
DnOP	85.7±8.79	85.2±7.49	92.9±8.64	83.1±9.08	89.4±6.75
DINP	94.4±6.86	93.6±7.98	101±7.47	-	-

Supplementary Table S14. The PAE recovery in the sum of particle-size fraction versus bulk soil in different layers of soils irrigated with wastewater (%).

Parameter			Value
IR	Maize	Adults	31000
		Children	19200
	Wheat	Adults	330000
		Children	204386
EF			365
ED		Adults	30
		Children	6
BW		Adults	65
		Children	16
AT			26280
RfD_o	DMP		10
	DEP		0.8
	DnBP		0.1
	BBP		0.2
	DEHP		0.02
	DnOP		0.04
SFO	BBP		0.0019
	DEHP		0.014

Supplementary Table S15. Parameters used in exposure carcinogenic and non-cancer risk assessments.

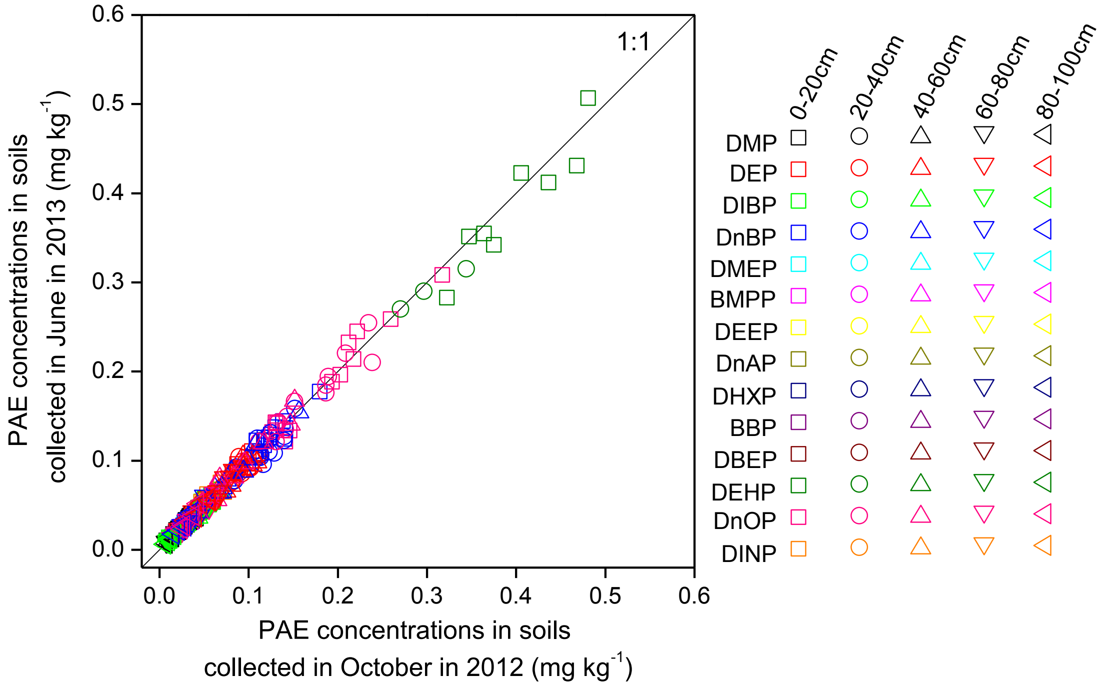

Supplementary Figure S1. The relationship of PAE concentrations in between soils collected in October 2012 and June 2013.

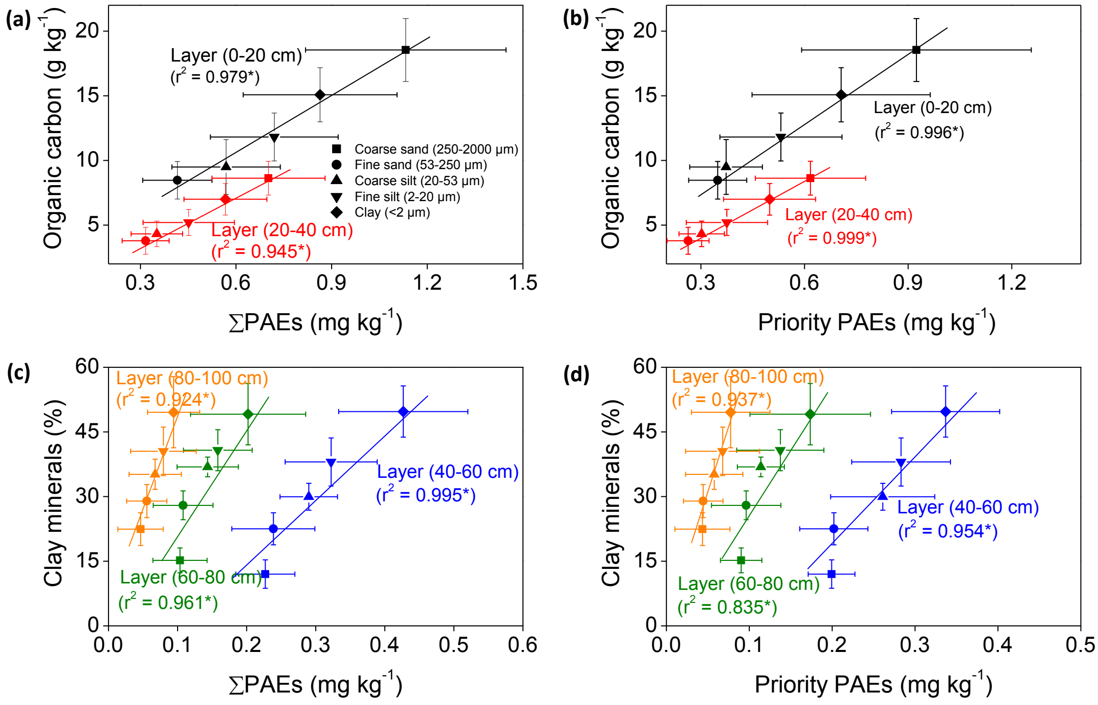

Supplementary Figure S2. Associations of concentrations of ΣPAE and six priority PAEs distributed in different soil particle-size fractions with organic carbon and clay minerals in different soil layers. An asterisk (*) indicates statistically significant ($P < 0.05$).

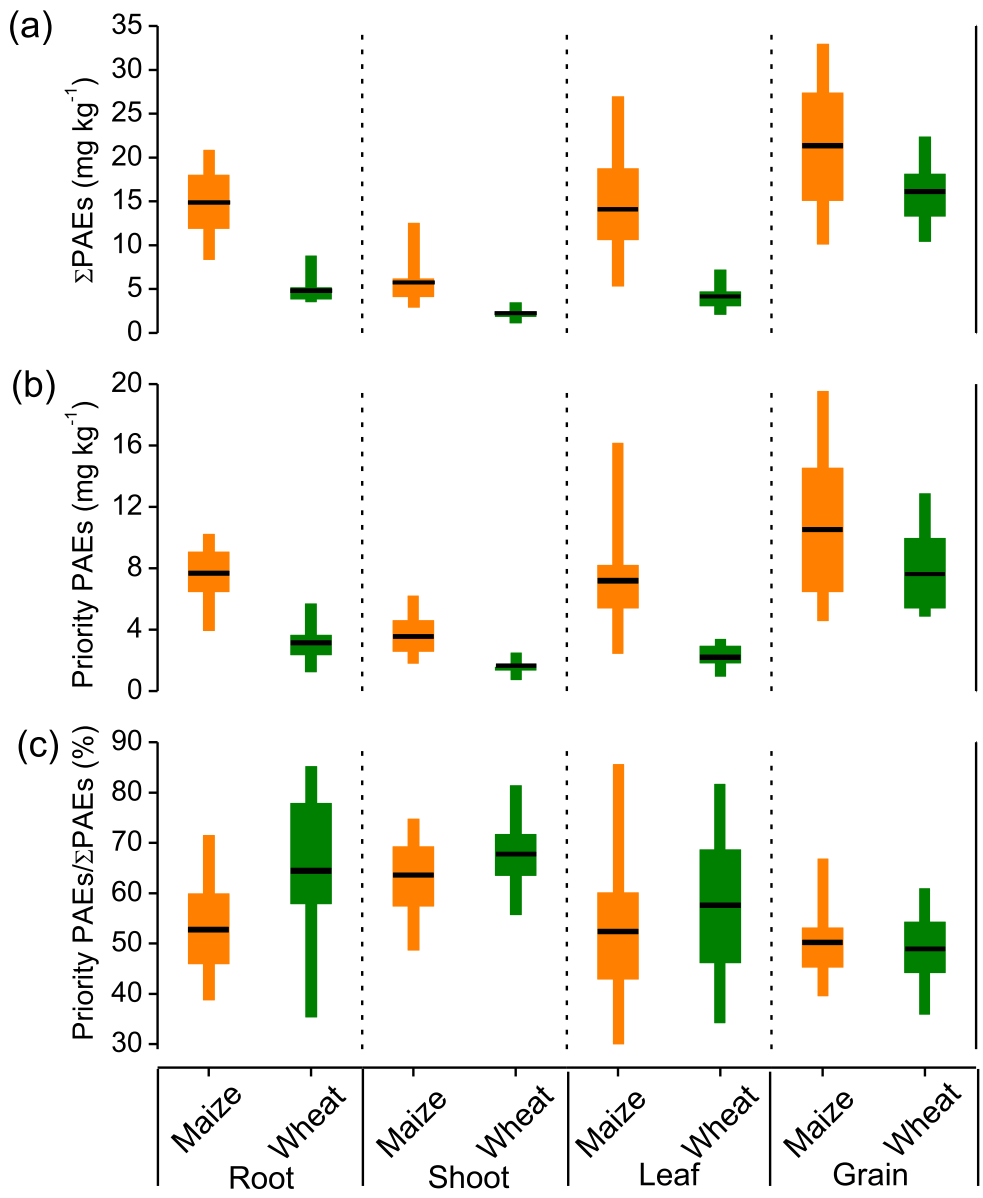

Supplementary Figure S3. Concentrations of ΣPAE (a), total concentrations of the six priority PAEs (b), and percentages of the six priority PAEs in ΣPAE (c) in maize and wheat tissues. Thin vertical line represents 10th and 90th percentiles, box represents 25th and 75th percentiles, and central horizontal line represents mean.

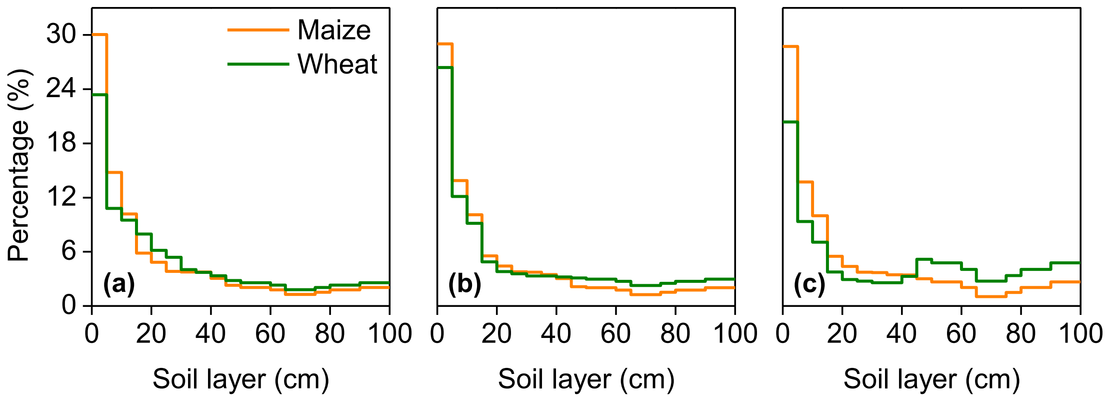

Supplementary Figure S4. Distribution proportion of root weight (a), length (b), and surface area (c) in soil profile.

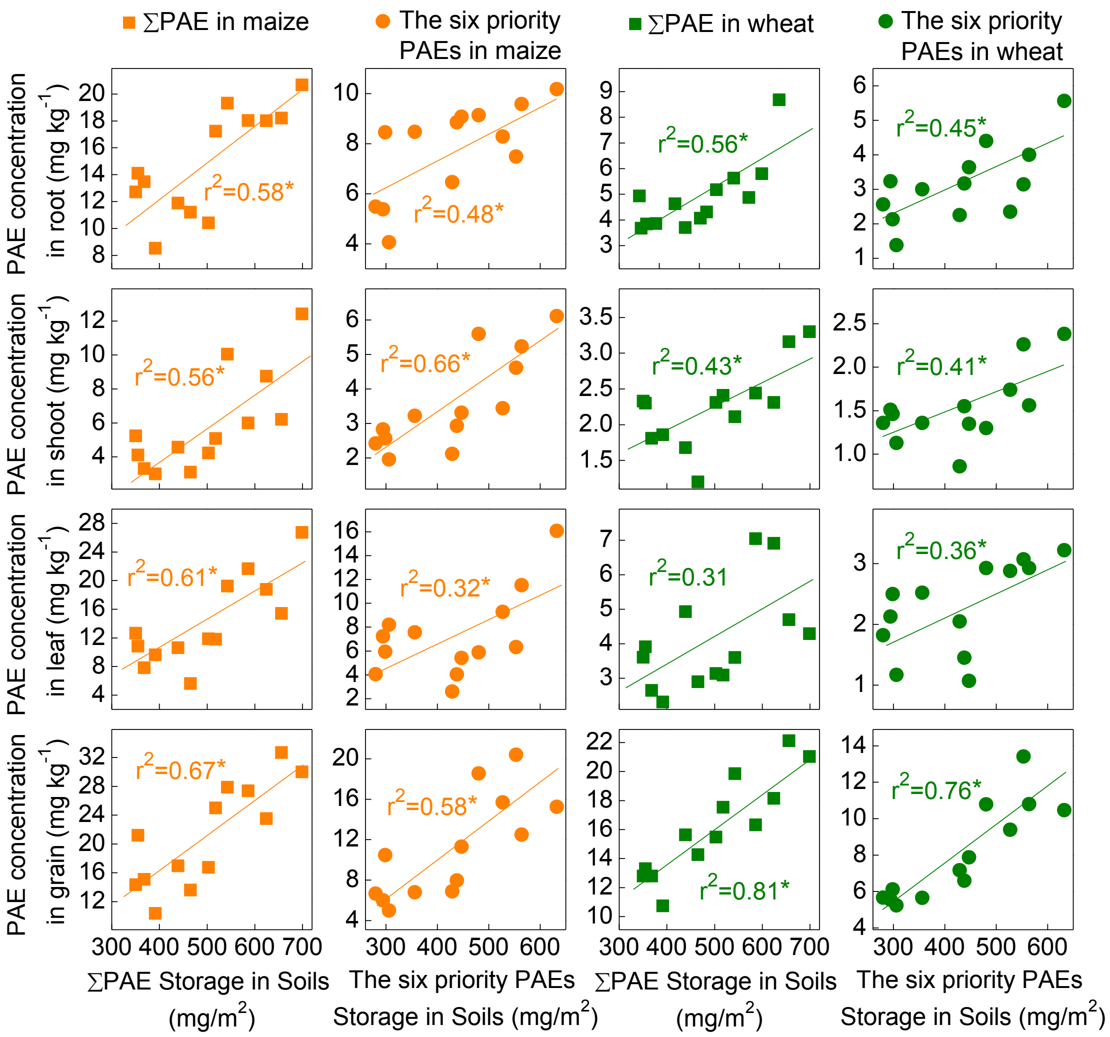

Supplementary Figure S5. Correlations between concentrations of ΣPAE and six priority PAEs in plant tissues and storage of ΣPAE and six priority PAEs in soils. An asterisk (*) indicates statistically significant ($P < 0.05$).

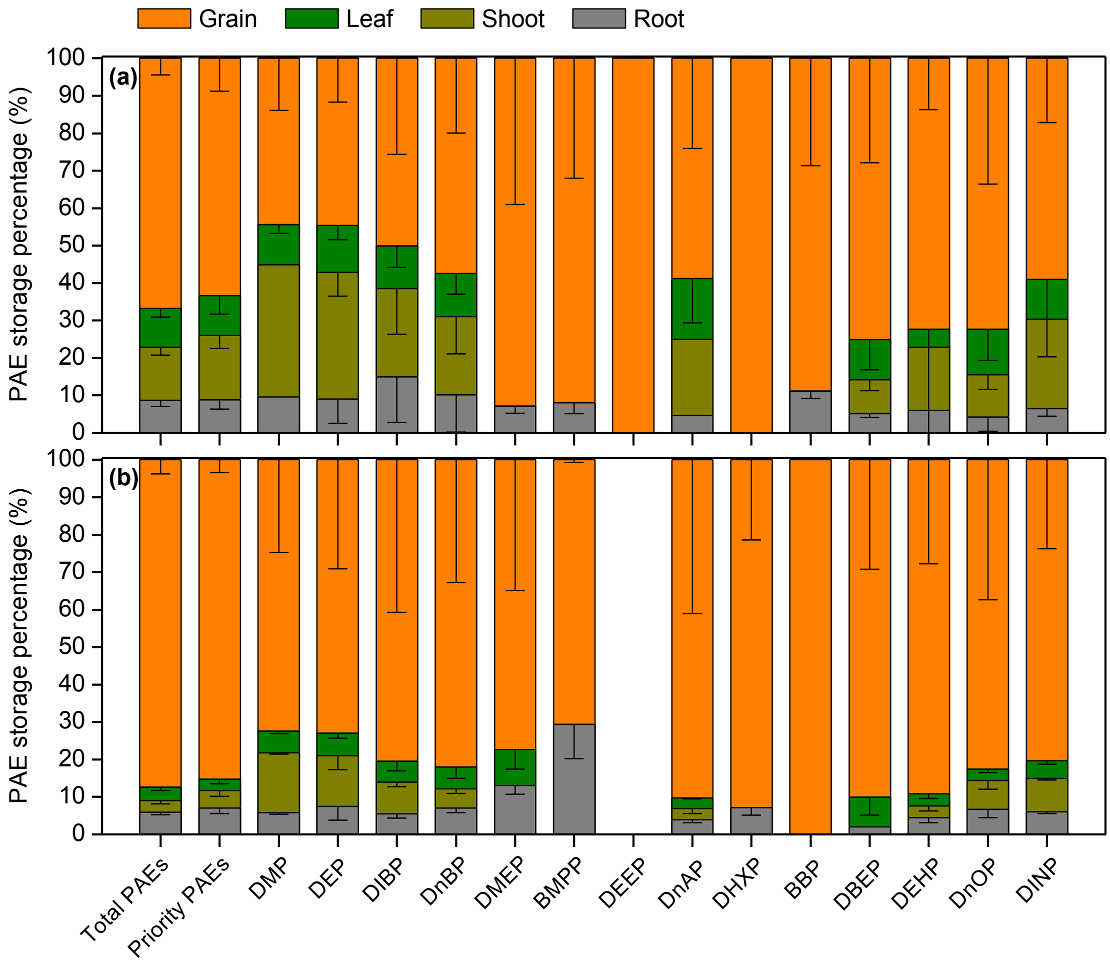

Supplementary Figure S6. Percentages of PAE storage distributed in different plant tissues in maize (a) and wheat (b).

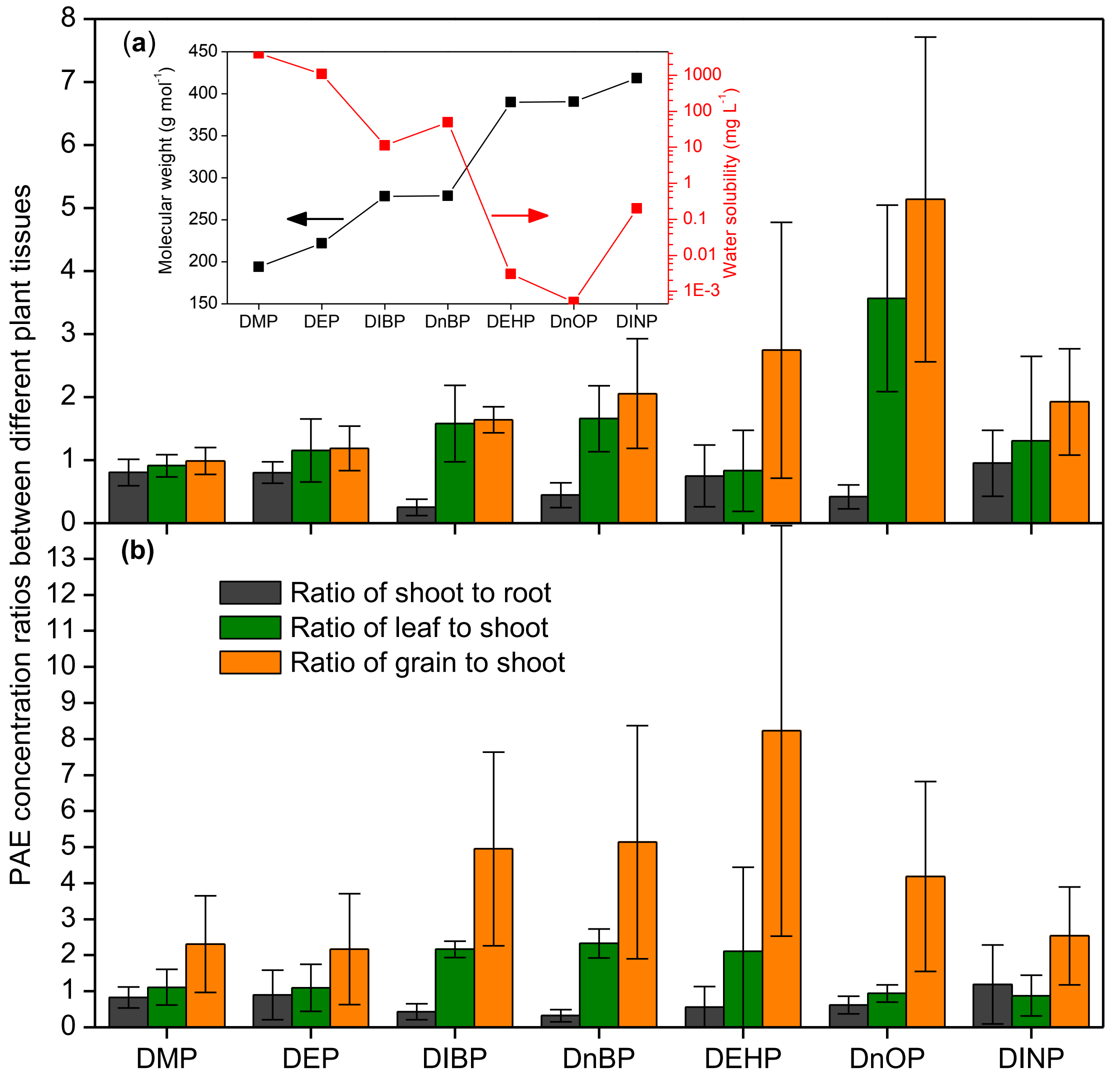

Supplementary Figure S7. PAE concentration ratios between different plant tissues of maize (a) and wheat (b). In (a), the inset shows the molecular weights and water solubilities of various PAE congeners.

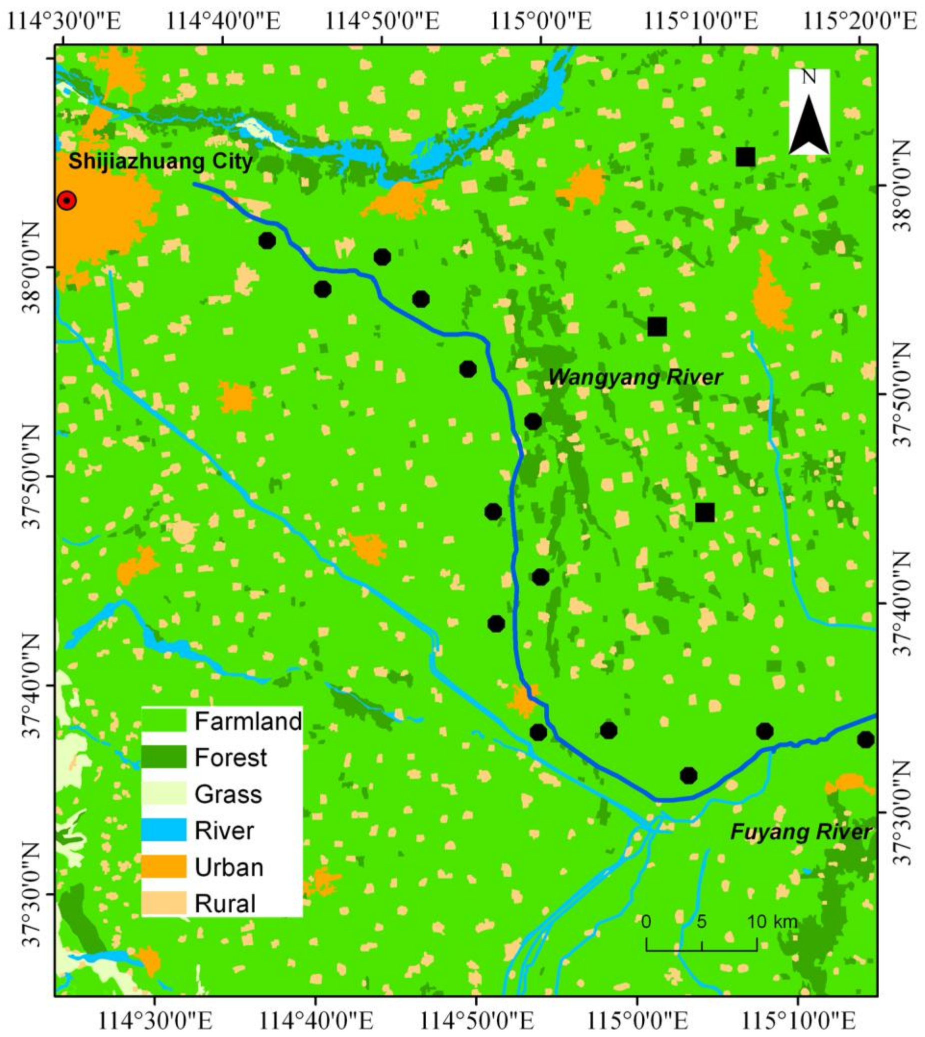

Supplementary Figure S8. Study area and sampling sites. Circles and squares indicate the sampling sites of the wastewater irrigated agricultural lands and the reference agricultural lands, respectively. (The map was created using ArcMap 10.2 http://www.esri.com/software/arcgis/arcgis-for-desktop).

References

1. Shanker, R., Ramakrishma, C. & Seth, P.K. Degradation of some phthalic acid esters in soil. *Environ. Poll. A* **39**, 1–7 (1985).
2. Russell, D.J., McDuffie, B. & Fineberg, S. The effect of biodegradation on the determination of some chemodynamic properties of phthalate esters. *J. Environ. Sci. Heal. A* **20**, 927–941 (1985).

3. Wang, J., Zhao, X. & Wu, W. Biodegradation of phthalic acid esters (PAEs) in soil bioaugmented with acclimated activated sludge. *Process Biochem*. **39**, 1837–1841 (2004).

4. Cousins, I.T., Mackay, D. & Parkerton, T.F. Physical-chemical properties and evaluative fate modeling of phthalate esters. In Staples, C. A., editors. Series Anthropogenic Compounds: Phthalate Esters. Springer Berlin Heidelberg, 57–84 (2003).

5. Chang, B.V., Lu, Y.S., Yuan, S.Y., Tsao, T.M. & Wang, M.K., Biodegradation of phthalate esters in compost-amended soil. *Chemosphere* **74**, 873–877 (2009).

6. Chen, Y. *et al.* Degradation of phthalic acid esters (PAEs) in soils. *Acta Scien. Cricum*. **17**, 340–345 (1997). (in Chinese)

7. Xu, G., Li, F. & Wang, Q. Occurrence and degradation characteristics of dibutyl phthalate (DBP) and di-(2-ethylhexyl) phthalate (DEHP) in typical agricultural soils of China. *Sci. Total Environ*. **393**, 333–340 (2008).

8. Wang, J., Liu, P., Shi, H. & Qian, Y. Biodegradation of phthalic acid ester in soil by indigenous and introduced microorganisms. *Chemosphere* **35**, 1747–1754 (1997).

9. Rüdel, H., Schmidt, S., Kördel, W. & Klein, W. Degradation of pesticides in soil: comparison of laboratory experiments in a biometer system and outdoor lysimeter experiments. *Sci. Total Environ.* **132**, 181–200 (1993).

10. Borgert, C.J., Roberts, S.M., Harbison, R.D. & James, R.C. Influence of soil half-life on risk assessment of carcinogens. *Regul. Toxicol. Pharm*. **22**, 143–151 (1995).

11. Roslev, P., Madsen, P.L., Thyme, J.B. & Henriksen, K. Degradation of phthalate and di-(2-ethylhexyl)phthalate by indigenous and inoculated microorganisms in

sludge-amended soil. *Appl. Environ. Microb*. **64**, 4711–4719 (1998).

12. Madsen, P.L., Thyme, J.B., Henriksen, K., Møldrup, P. & Roslev, P. Kinetics of di-(2-ethylhexyl)phthalate mineralization in sludge-amended soil. *Environ. Sci. Technol*. **33**, 2601–2606 (1999).
